# Deep Phenotyping of Headache in Hospitalized COVID-19 Patients *via* Principal Component Analysis

**DOI:** 10.3389/fneur.2020.583870

**Published:** 2020-12-17

**Authors:** Álvaro Planchuelo-Gómez, Javier Trigo, Rodrigo de Luis-García, Ángel L. Guerrero, Jesús Porta-Etessam, David García-Azorín

**Affiliations:** ^1^Imaging Processing Laboratory, Universidad de Valladolid, Valladolid, Spain; ^2^Headache Unit, Department of Neurology, Hospital Clínico Universitario de Valladolid, Valladolid, Spain; ^3^Neuroscience Research Unit, Institute for Biomedical Research of Salamanca, Salamanca, Spain; ^4^Department of Medicine, Universidad de Valladolid, Valladolid, Spain; ^5^Headache Unit, Department of Neurology, Hospital Clínico San Carlos, Madrid, Spain

**Keywords:** COVID-19, headache disorders, migraine, tension-type headache, machine learning

## Abstract

**Objectives:** Headache is a common symptom in systemic infections, and one of the symptoms of the novel coronavirus disease 2019 (COVID-19). The objective of this study was to characterize the phenotype of COVID-19 headache *via* machine learning.

**Methods:** We performed a cross-sectional study nested in a retrospective cohort. Hospitalized patients with COVID-19 confirmed diagnosis who described headache were included in the study. Generalized Linear Models and Principal Component Analysis were employed to detect associations between intensity and self-reported disability caused by headache, quality and topography of headache, migraine features, COVID-19 symptoms, and results from laboratory tests.

**Results:** One hundred and six patients were included in the study, with a mean age of 56.6 ± 11.2, including 68 (64.2%) females. Higher intensity and/or disability caused by headache were associated with female sex, fever, abnormal platelet count and leukocytosis, as well as migraine symptoms such as aggravation by physical activity, pulsating pain, and simultaneous photophobia and phonophobia. Pain in the frontal area (83.0% of the sample), pulsating quality, higher intensity of pain, and presence of nausea were related to lymphopenia. Pressing pain and lack of aggravation by routine physical activity were linked to low C-reactive protein and procalcitonin levels.

**Conclusion:** Intensity and disability caused by headache attributed to COVID-19 are associated with the disease state and symptoms. Two distinct headache phenotypes were observed in relation with COVID-19 status. One phenotype seems to associate migraine symptoms with hematologic and inflammatory biomarkers of severe COVID-19; while another phenotype would link tension-type headache symptoms to milder COVID-19.

## Introduction

Headache is one of the most common symptoms in coronavirus disease 2019 (COVID-19) (1, 2). However, differently to other symptoms, as anosmia or myalgia, the headache phenotype appears to be non-uniform (3). The most reported phenotype is bilateral pain, pulsating or pressing quality in temporoparietal or frontal region, with moderate to severe intensity (4).

The clinical presentation of COVID-19 is linked to its pathophysiology. Interferon gamma type I-III seem related to general systemic symptoms, such as fever and myalgia, among others. The cytokine release, macrophage activation and lymphocyte depletion are related with endothelial dysfunction and micro-thrombosis (5). In addition, the downregulation of type 2 angiotensin II receptors and the upregulation of type 1 angiotensin II receptors causes vasoconstriction and a proinflammatory state (6). The laboratory correlate to COVID-19 pathophysiology is observed by lymphopenia and increased C-reactive protein (CRP), procalcitonin (PCT) and D-dimer, among others (7). In patients with COVID-19 and headache, there was at least one abnormal laboratory value in the first measure, being the most common abnormal increased CRP levels (8).

The objective of this study was to analyze whether the headache phenotype did correlate with the laboratory biomarkers that have been linked to COVID-19 pathophysiology and/or the COVID-19 clinical presentation, by performing an analysis based on machine learning techniques.

## Materials and Methods

We conducted a cross-sectional study nested in cohort of patients. The Ethics Review Board of Valladolid East health area approved the study (PI: 20-1738).

### Participants

The inclusion criteria for participants were: (1) Confirmed COVID-19 diagnosis by real time reverse-transcriptase-polymerase-chain-reaction (RT-PCR) assay from respiratory tract samples, or by the presence of anti-SARS-CoV-2 IgM + IgA antibodies in patients with clinical symptoms, according to the World Health Organization protocols (9, 10); (2) suffering from headache throughout the course of COVID-19; (3) hospitalization because of COVID-19; (4) agreement to participate; (5) fulfillment of criteria for acute headache attributed to systemic viral infection according to the International Classification of Headache Disorders, 3rd edition (ICHD-3) (11).

The exclusion criteria were: (1) Acute secondary headache with better agreement for a diagnosis different to acute headache attributed to systemic viral infection according to the ICHD-3 (11); (2) death during the hospitalization; (3) previous dementia or cognitive impairment that made difficult a detailed description of the suffered headache; (4) poor medical condition that difficulted the precise description of the headache phenotype; (5) no answer to the invitation to participate in the study; (6) rejection to take part in the study.

All the patients admitted to the Hospital Clínico Universitario de Valladolid (Valladolid, Spain) from March 8th to April 11th, 2020 were screened. The information employed in this study was collected from the primary care electronic digital records, the emergency room records and the hospitalization reports. Every patient was asked about suffering from headache, and those patients who answered with a positive response were invited to take part in the study. Two neurologists with expertise in headache medicine interviewed the patients according to a pre-specified structured interview.

### Variables

Six groups of variables related to headache were analyzed as variables of interest: (1) Intensity of the pain (0–10 numeric rating scale; 0: no pain, 10: maximum intensity); (2) disability caused by headache, self-rated by the patient (0–100 numeric rating scale; 0: no disability, 100: complete disability); (3) presence of typical migraine and tension-type headache (TTH) features (categorical variables showing presence of each symptom included in criteria C and D for migraine without aura from the ICHD-3, and ICHD-3 criteria C and D for TTH, analyzing if patients fulfilled the ICHD-3 criteria); (4) laterality (categorical variables indicating unilateral or bilateral pain); (5) topography (categorical variables for presence of pain in diverse areas, e.g., frontal); (6) quality of the pain (e.g., pressing). For the topography and quality of pain, patients were asked to describe the predominant one. The cranial territories or phenotypic characteristics present at least in 20% of the subjects were included, taking into account the number of possible regions, with no consideration of simultaneous pain in more than one region.

As main independent variables, diverse COVID-19 symptoms (categorical variables describing the presence of each different symptom) and results from laboratory tests were evaluated. The COVID-19 symptoms included in this study were arthralgia, chest pain, cough, diarrhea, dyspnea, expectoration, fatigue, fever, headache (100% in this sample), hyposmia or anosmia, lightheadedness, myalgia, nausea, odynophagia, rhinorrhea, skin rash, weakness, and vomiting. We gathered different laboratory parameters on admission and worst values during the stay. These values were represented as numerical variables and categorical variables indicating abnormal levels. Reference values and units are detailed in Supplementary Table 1. Other analyzed independent variables were demographic (age and sex), prior medical history (more details in Supplementary Table 1), previous history of headache (including a 0–100 rating scale to assess the level of similarity), modified Rankin scale, other symptoms associated with the headache (cranial autonomic symptoms, hypersensitivity to stimuli, vegetative symptoms, and aura), other headache features (duration, pain during sleep, worst experienced headache, aggravation by walking, head or ocular movements, and clinophilia), presence of pneumonia (either diagnosed *via* chest X-ray or computed tomography), and variables related to treatment (employed treatments and resistance to treatment).

### Statistical Analysis and Principal Component Analysis

First, univariate analyses were performed. The continuous variables, intensity of pain and disability caused by headache, were analyzed using Generalized Linear Models (GLM) with a Gaussian distribution. The remaining binary variables related to headache were analyzed using GLM with a binomial family, i.e., logistic regression models. Considering the high number of possible binary variables which could be analyzed, only the most frequent topography, quality, and criteria C and D from the ICHD-3 for migraine were examined.

To detect specific patterns between the variables, a Principal Component Analysis (PCA) of mixed data (continuous and categorical) was carried out. For the main continuous variables, different PCA were performed for each group of categorical main variables. In the case of the categorical laboratory tests and COVID-19 symptoms variables, the variables with statistically significant results in the univariate analyses were introduced in the PCA. Furthermore, for the main categorical variables, PCA using the significant continuous results from laboratory tests that were statistically significant in the univariate analyses were carried out on the one hand, and CRP, PCT, lymphocytes, and D-dimer (worst and first values on admission) were analyzed on the other hand. Furthermore, the most frequent characteristics found in this sample (8, 12), i.e., hypersensitivity to stimuli, pressing and intense pain, and pain in the frontal area (which was also bilateral diffuse pain in most cases), were also assessed.

Finally, for the response variables analyzed with univariate GLM, a multivariate model was obtained. Only variables with *p*-value equal or lower than 0.20 in the univariate analyses and with no or very low amount of missing values (<5%) were considered to be introduced in the multivariate model. The Akaike's Information Criterion (lowest value) was used to obtain the final multivariate GLM in combination with an automatic stepwise (use of forward and backward steps) procedure. To reduce effects of overfitting related to a great number of employed variables, the variable with the highest *p*-value was removed from the model until the model showed no excessive overfitting effects, i.e., no exaggerated standard error values. A false discovery rate (FDR) procedure was used to correct for multiple comparisons in the multivariate models (13).

A *p*-value of 0.05 was considered as statistical signification threshold. Complete-case analysis was employed in situations with variables with missing values. There was no previous estimation of sample size because the initial objective of the analysis with the patients was exploratory. R statistical software version 3.5.2 was used for the analysis.

## Results

After the examination of inclusion and exclusion criteria, 106 patients were included in the study. The number of hospitalized patients because of COVID-19 with a positive test was 580, and among them, 136 patients described headache. A complete flow diagram is shown in Supplementary Figure 1.

The mean age of the sample was 56.6 ± 11.2 years, with 76 patients (71.7%) older than 49, and 68 women (64.2%) participated in the study. Regarding the previous history of headache, 61 patients presented a previous history of headache (57.5%). Among these patients, 28 suffered previously from TTH, 16 from migraine, two from both TTH and migraine, and 15 from other headache (e.g., cervicogenic headache, hypnic headache, and episodic cluster headache). Taking into account that the previous history of the diverse types of headache disorders may be related to the headache phenotype, the results associated with this variable are reported in the following subsections. In the subjects with prior history of headache, the level of similarity to prior headache (0–100 rating scale) was 32.5 ± 29.5 (median = 40, interquartile range = 0–50).

Criterion C for migraine from the ICHD-3 was fulfilled by 55 patients (51.9%), and criterion D by 40 patients (37.7%). Criterion C for TTH from the ICHD-3 was fulfilled by 90 patients (84.9%), and criterion D by 66 patients (62.3%). The most frequent quality of pain was pressing, which was present in 80 patients (75.4%), and the most frequent location was frontal, which was present in 88 patients (83.0%). Both characteristics were analyzed in uni- and multivariate GLM.

### PCA and Univariate Models—Continuous Variables

Significant results from univariate models and from PCA for intensity of headache and self-reported disability are detailed in Supplementary Tables 2, 3, respectively, and in the following subsections. Regarding the previous history of headache disorders, we found no significant associations with either intensity of headache or self-reported disability caused by headache. However, we found that lower values of intensity and disability scores were significantly related to higher similarity to prior headache in the univariate models (Supplementary Table 2).

#### COVID-19 Symptoms and Laboratory Tests

The variables that showed statistically significant association in the univariate disability models were lightheadedness and fever, and abnormal platelet values on admission and during hospitalization (qualitative variables), and therefore those variables were included in the PCA analysis. In the univariate models, higher disability score was associated with fever, while lower disability score was associated with lightheadedness.

**Figure 1:**
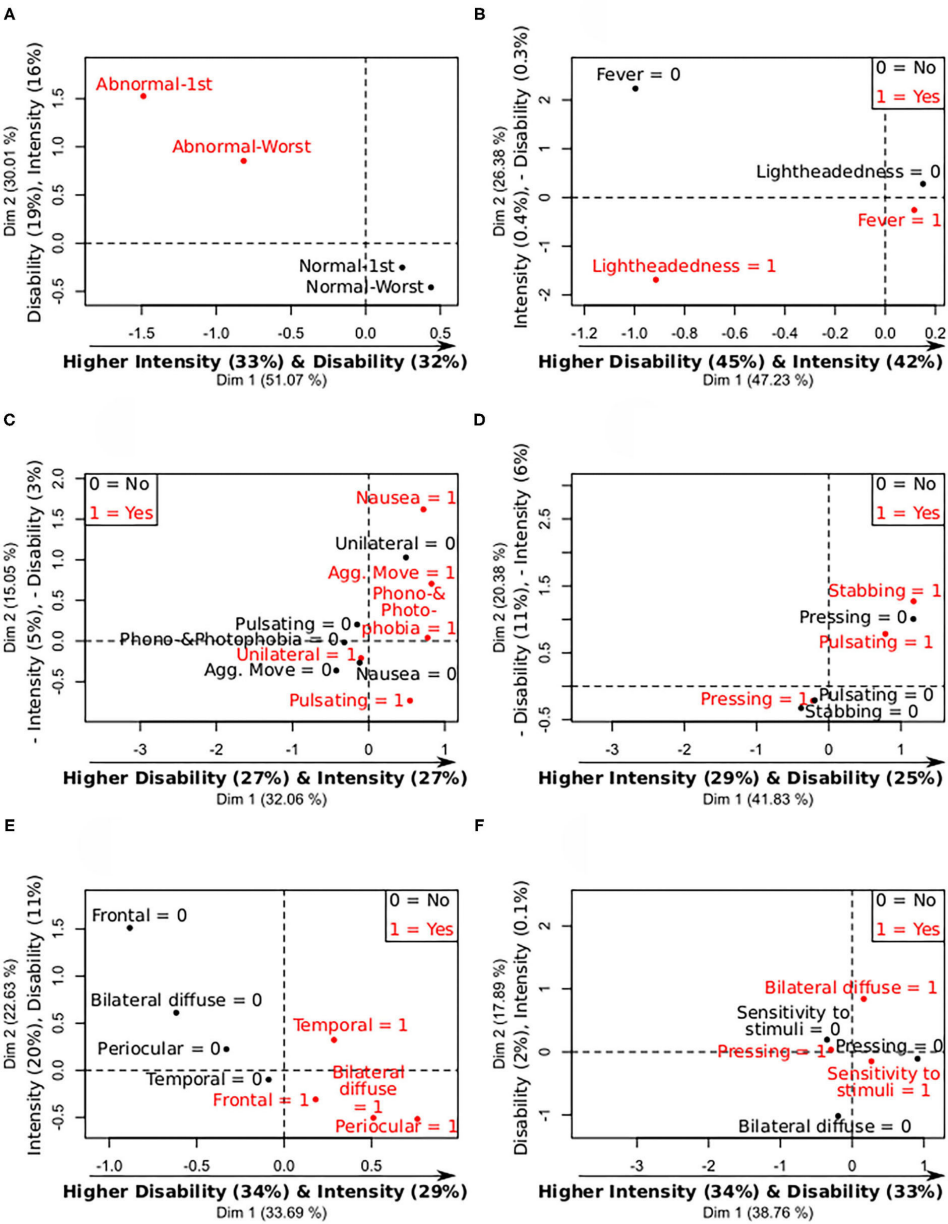
Summary of mix PCA of headache intensity and disability caused by headache (continuous variables), and the analyzed categorical features. X- and Y-axis contain the values of the first and second principal components, and the relative contribution of each continuous variable to the component, remarking in bold the most important relationships between the continuous variables and the components. 0 = no symptom; 1 = present symptom. **(A)** Levels—Platelets intensity and disability. **(B)** Levels—COVID-19 symptoms intensity and disability. **(C)** Levels—Migraine symptoms intensity and disability. **(D)** Levels—Quality of pain intensity and disability. **(E)** Levels—Laterality and topography intensity and disability. **(F)** Levels—Most frequent symptoms intensity and disability.

The PCA showed three groups clearly differentiated, one of them composed by people with fever and lightheadedness, other by people with only fever, and the last group by people with neither fever nor lightheadedness. Regarding laboratory tests, three clearly differentiated patterns of intensity and disability were also observed. The first group exhibited low disability and low intensity of headache, that had abnormal platelet count on admission and during the hospitalization; the second group had higher intensity and disability than the former and normal platelet values on admission, but not during the hospitalization. The last group had the highest intensity and disability and had a normal platelet count, both on admission and during hospitalization. These PCA results showing the intensity and disability differences between the abnormal and normal levels are depicted in Figure 1A and Supplementary Figure 2.

With respect to COVID-19 symptoms, people with lightheadedness and fever presented the lowest (negative mean) PCA second component values, and people with only fever exhibited medium values. Higher second PCA component values were associated with higher intensity and with lower disability. The higher disability was observed in the two groups of patients that had fever as COVID-19 symptom. The PCA results showing the intensity and disability differences between patients with and without fever and lightheadedness can be seen in Figure 1B and Supplementary Figure 2. The group with fever and lightheadedness presented the lowest headache intensity values, and the group with fever but no lightheadedness had the highest intensity values.

#### Migraine and TTH Symptoms

Fulfillment of criterion C of TTH was significantly associated with lower intensity and disability scores, while migraine symptoms such as pulsating pain, aggravation by routine physical activity, and simultaneous phonophobia or photophobia, were associated with higher intensity and disability scores. The last two positive associations are equivalent to a negative association with the absence of the features (e.g., no aggravation by physical activity), which is in line with lower intensity and disability values in TTH.

The PCA did not reveal clearly differentiated groups based on migraine symptoms, in contrast to the analyses with COVID-19 symptoms and laboratory tests. However, noticeable differences can be observed between people with nausea and/or aggravation caused by routine physical activity, and no unilateral pain. These differences can be appreciated in Figure 1C and Supplementary Figure 3. Better clustering was found with fulfillment of criteria for migraine and TTH from the ICHD-3 (Supplementary Figure 4).

#### Quality of Pain

Pulsating and stabbing pain were associated with higher values of intensity of headache, while pressing pain was associated with lower values of intensity and disability caused by headache. Although stabbing pain was present in <20% of the subjects (15 subjects, 14.2%), it was finally included in the PCA because of the significant association.

In line with the intensity and disability univariate models, people with pressing pain presented different PCA scores in comparison with people with pulsating or stabbing pain, as can be seen in Figure 1D and Supplementary Figure 5.

#### Laterality and Topography of Pain

A significant association was found between higher values of disability caused by headache and bilateral diffuse pain. The PCA revealed a clear separation based on frontal pain. Approximately two thirds of the subjects with frontal pain presented bilateral diffuse pain, and one third periocular pain. No clear distinctions were found for temporal pain. The results from this simultaneous analysis comparing intensity and disability between diverse areas are shown in Figure 1E and Supplementary Figure 6.

#### Most Frequent Characteristics

Bilateral diffuse pain was the characteristic showing the higher difference in the PCA comparing bilateral diffuse pain, pressing pain and hypersensitivity to stimuli, as can be seen in Figure 1F and Supplementary Figure 7.

### PCA and Univariate Models—Categorical Main Variables

Significant univariate models and PCA results are shown in Supplementary Tables 4–8.

#### Migraine Symptoms

With respect to the univariate models analyzing criteria C and D of migraine from the ICHD-3 and their relationship with laboratory tests, a significant negative association (higher values, lower odds of presenting a phenotype with migraine features) was found with worst levels and levels on admission of lactate dehydrogenase (LDH) and glomerular filtration. There were no higher values of any continuous variable associated with higher odds of presenting headache with migraine features. No significant association was found between criteria C and D of migraine, and previous history of headache. The PCA showed that the first component, which explained higher variance, was weighted mostly by glomerular filtration (Supplementary Table 5).

**Figure 2:**
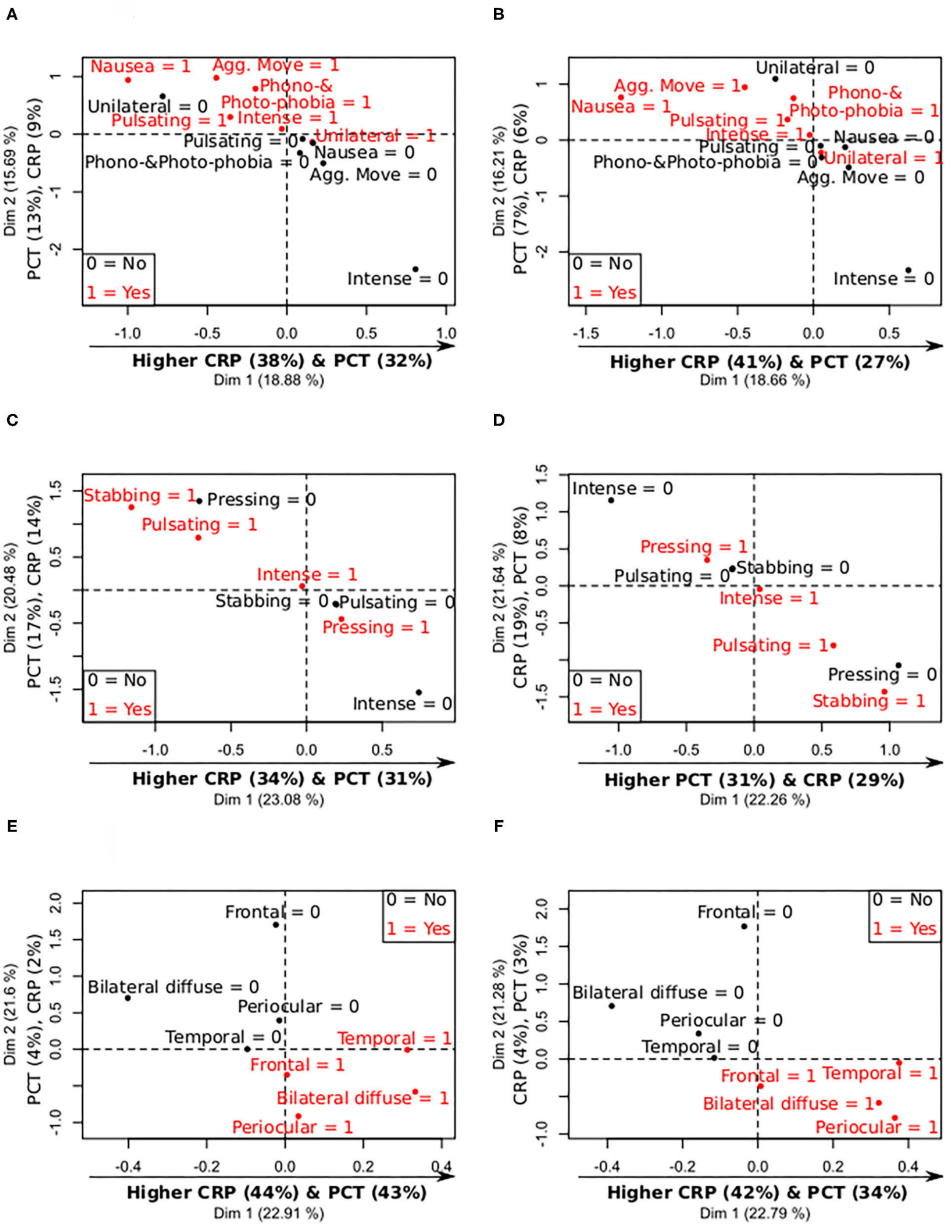
Summary of mix PCA of CRP, PCT, D-dimer, and lymphocytes levels (continuous variables), and the analyzed categorical features, focused on CRP and PCT levels. X- and Y-axis contain the values of the first and second principal components, and the relative contribution of each continuous variable to the component, remarking in bold the most important relationships between the continuous variables and the components. 0 = no symptom; 1 = present symptom. **(A)** Levels—Migraine symptoms CRP and PCT (admission). **(B)** Levels—Migraine symptoms CRP and PCT (worst values). **(C)** Levels—Quality of pain CRP and PCT (admission). **(D)** Levels—Quality of pain CRP and PCT (worst values). **(E)** Levels—Laterality and topography CRP and PCT (admission). **(F)** Levels—Laterality and topography CRP and PCT (worst values).

**Figure 3:**
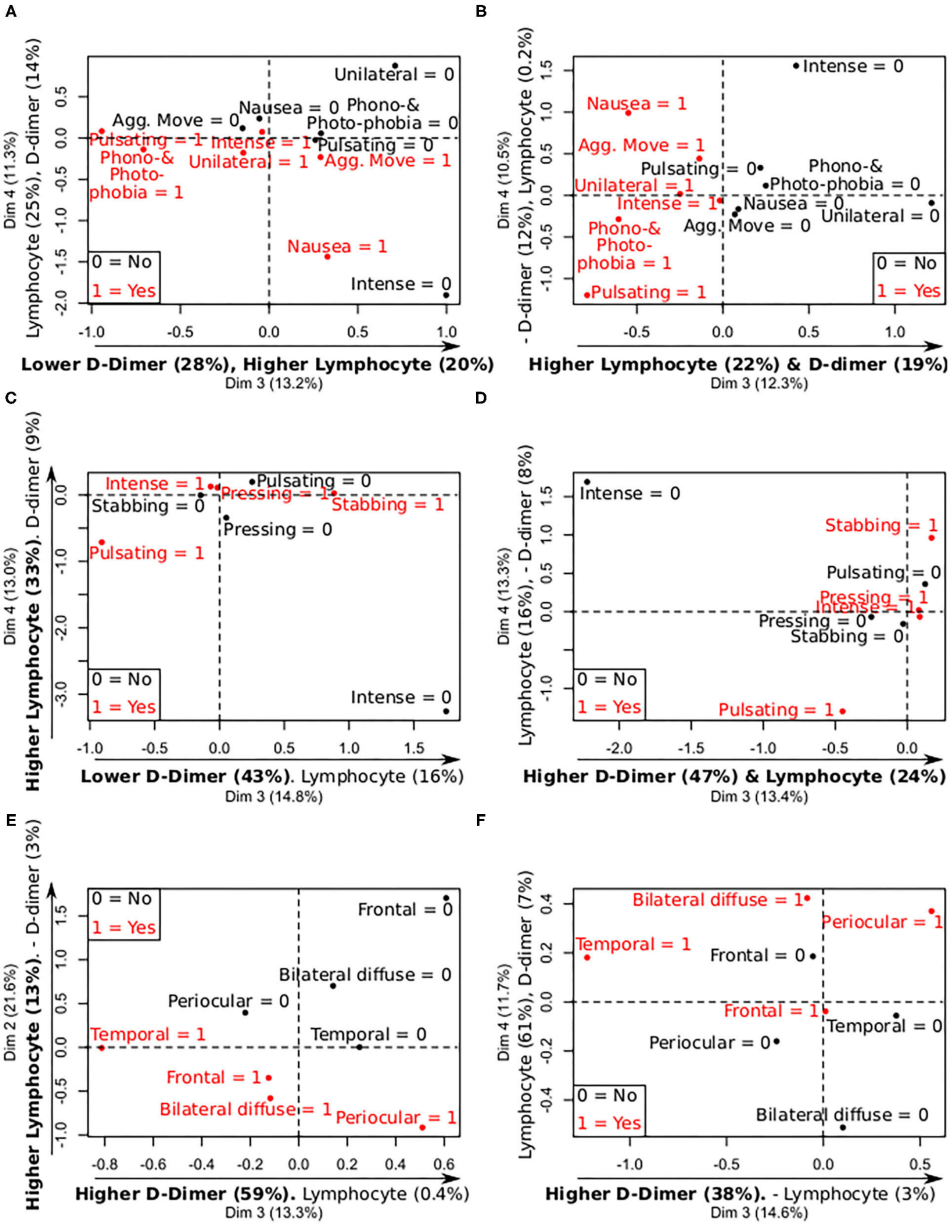
Summary of mix PCA of CRP, PCT, D-dimer, and lymphocytes levels (continuous variables), and the analyzed categorical features, focused on D-dimer and lymphocyte levels. X- and Y-axis contain the values of two principal components, and the relative contribution of each continuous variable to the component, remarking in bold the most important relationships between the continuous variables and the components. 0 = no symptom; 1 = present symptom. **(A)** Levels—Migraine symptoms D-dimer and lymphocytes (admission). **(B)** Levels—Migraine symptoms D-dimer and lymphocytes (worst values). **(C)** Levels—Quality of pain D-dimer and lymphocytes (admission). **(D)** Levels—Quality of pain D-dimer and lymphocytes (worst values). **(E)** Levels—Laterality and topography D-dimer and lymphocytes (admission). **(F)** Levels—Laterality and topography D-dimer and lymphocytes (worst values).

According to the PCA with the CRP and PCT values on admission and worst levels, the symptoms with the highest differences were nausea (lower CRP-values) and aggravation by movement (higher PCT-values), as can be observed in Figures 2A,B and Supplementary Figure 8. According to the lymphocyte and D-dimer levels, patients with nausea presented lower D-dimer and lymphocyte values on admission and worst levels, and patients with pulsating pain showed lower lymphocyte count, as can be seen in Figures 3A,B and Supplementary Figure 8.

The PCA analyzing LDH and glomerular filtration (four continuous variables) and migraine symptoms, suggested that patients with headache aggravation by physical activity showed lower PC1 values (weighted mostly by glomerular filtration), as shown in Supplementary Figure 8.

#### Quality of Headache

No significant association was found between quality of headache and continuous results from laboratory tests in the univariate models. The same lack of statistically significant association was also found between pressing pain and previous history of headache. In the PCA using the CRP, PCT, lymphocyte, and D-dimer values, a clear separation was observed in patients with pressing compared to stabbing and pulsating pain based on lower values on admission and worst levels of CRP and PCT, as can be seen in Figures 2C,D and Supplementary Figure 9. Additionally, pulsating pain was associated with lower lymphocyte count on admission and worst values, as illustrated in Figures 3C,D and Supplementary Figure 9.

#### Topography of Headache

Lower leukocyte, lymphocyte and platelet levels on admission, and worst platelet levels, were significantly associated with laterality-topography of the pain. The PCA with the previous variables showed differences based on frontal pain and bilateral diffuse pain, which can be seen in Supplementary Figure 10, obtaining similar results with respect to results shown in Supplementary Figure 6. No significant association was found between pain in the frontal area and previous history of headache.

In the PCA using the CRP, PCT, lymphocyte and D-dimer values, higher CRP and PCT values on admission, and worst values, were clearly differentiated in patients with temporal and bilateral diffuse pain, as shown in Figures 2E,F and Supplementary Figure 11. Lower lymphocyte count, on admission and worst values, was observed in patients with frontal and bilateral diffuse pain, and lower D-dimer values in patients with temporal pain, as can be seen in Figures 3E,F and Supplementary Figure 11. In Figure 3F, lymphocyte count seems higher in patients with bilateral diffuse pain, but it is worth noting that the second component (Figure 2F) is weighted mainly by the lymphocyte count (8% compared to 4–3% of CRP and PCT), and patients with bilateral diffuse pain presented lower PC2 values.

#### PCA: Most Frequent Characteristics and Laboratory Tests

**Figure 4:**
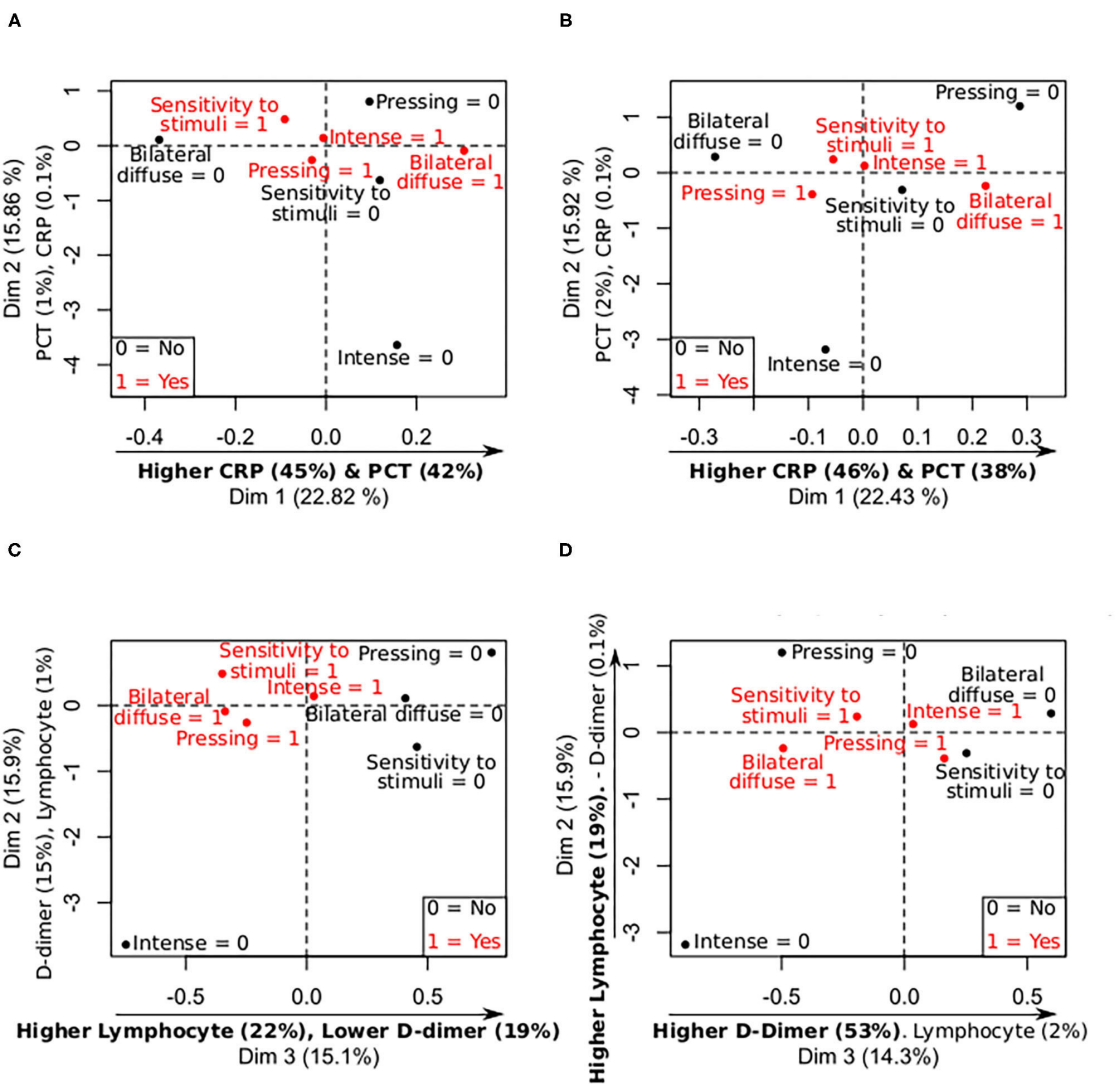
Summary of mix PCA of CRP, PCT, D-dimer, and lymphocytes levels (continuous variables), and most frequent symptoms in the sample (categorical variables). X- and Y-axis contain the values of two principal components, and the relative contribution of each continuous variable to the component, remarking in bold the most important relationships between the continuous variables and the components. 0 = no symptom; 1 = present symptom. **(A)** Levels—Most frequent symptoms CRP and PCT (admission). **(B)** Levels—Most frequent symptoms CRP and PCT (worst values). **(C)** Levels—Most frequent symptoms D-dimer and lymphocytes (admission). **(D)** Levels—Most frequent symptoms D-dimer and lymphocytes (worst values).

As mentioned in previous subsections, PCA results showed that bilateral diffuse pain was associated with higher CRP and PCT values on admission and worst levels, and with lower values for pressing pain. Hypersensitivity to stimuli was associated with higher CRP and PCT values, as depicted in Figures 4A,B and Supplementary Figures 12, 13. Lower lymphocyte count on admission and worst values was observed in bilateral diffuse pain, as mentioned in previous subsections, which can be also seen in Figures 4C,D. No other clear trend was observed.

### Multivariate Models

#### Intensity and Disability

After correcting for multiple comparisons, lower values of intensity of headache were significantly associated with resistance to treatment. Higher intensity values were significantly associated with female sex, aggravation by physical activity, and pulsating or stabbing quality compared to pressing quality. The complete multivariate model is shown in Table 1. In this table, coefficients reflect that, for example, people whose headache was aggravated by physical activity, had 2.13 more intensity points (on average) that people with no aggravation.

**Table 1:** Multivariate GLM of the headache intensity in patients hospitalized because of COVID-19.

	**Coefficient and 95% CI**	**Unadjusted *p*-value**	**Adjusted *p*-value (FDR)**
Intensity model			
Independent term	3.70 (1.84, 5.55)	<0.001	0.001
**Treatment resistant**	−0.74 (−1.29, −0.19)	0.010	0.032
**Sex (Female vs. Male)**	1.18 (0.65, 1.72)	<0.001	<0.001
**Aggravation by physical activity**	2.13 (0.47, 3.79)	0.014	0.039
Quality of headache			
**Pulsating vs. pressing**	2.06 (1.09, 3.03)	<0.001	<0.001
**Stabbing vs. pressing**	1.60 (0.60, 2.59)	0.002	0.010
Aggravation by head movement	−1.37 (−2.96, 0.23)	0.096	0.11
Lightheadedness	−0.76 (−1.49, −0.03)	0.044	0.076
Abnormal platelets on admission	−0.71 (−1.44, 0.01)	0.058	0.076
High blood pressure comorbidity	−0.63 (−1.18, −0.08)	0.029	0.069
Progressive	0.54 (−0.14, 1.22)	0.12	0.13
Headache as first COVID-19 symptom	0.61 (−0.00, 1.22)	0.054	0.076
Phonophobia and photophobia	0.62 (0.02, 1.23)	0.047	0.076
Expectoration	0.82 (0.07, 1.56)	0.034	0.073
TTH—Criterion C	0.87 (−0.11, 1.85)	0.086	0.10
Pathologic chest X-Ray	1.37 (0.02, 2.73)	0.050	0.076
Disability model			
Independent term	12.36 (−9.53, 34.24)	0.27	0.27
**Lightheadedness**	−14.17 (−24.81, −3.53)	0.011	0.030
**Abnormal platelet count on admission**	−12.53 (−22.85, −0.20)	0.020	0.042
**Treatment resistant**	−9.99 (−17.84, −2.13)	0.015	0.036
**Aggravation by physical activity**	12.16 (4.26, 20.06)	0.003	0.029
**Phonophobia and photophobia**	12.78 (3.97, 21.60)	0.006	0.030
**Sex (Female vs. Male)**	14.03 (5.36, 22.70)	0.002	0.029
**Fever**	14.65 (2.10, 27.20)	0.025	0.047
**Expectoration**	14.88 (3.98, 25.79)	0.009	0.030
**Leukocyte count on admission**	0.0020 (0.0005, 0.0034)	0.010	0.030
Smoking (previous or current)	−10.59 (−23.49, 2.31)	0.11	0.13
Use of Angiotensin-converting-enzyme inhibitors and angiotensin II receptors	−7.66 (−16.29, 0.98)	0.086	0.11
Days from admission to worst lymphocyte count	−0.60 (−1.21, 0.01)	0.056	0.080
Neurological symptoms of headache	8.51 (−2.00, 19.01)	0.12	0.13
Bilateral diffuse pain	8.21 (0.54, 15.88)	0.039	0.060
**Quality of headache**			
Pulsating vs. pressing	12.93 (1.24, 24.62)	0.033	0.056
Stabbing vs. pressing	10.53 (−2.82, 23.87)	0.13	0.13

*The bold terms show statiscally significant variables after the FDR correction (if a p-value is provided for the variable) or indicate a specific model*.

With respect to the disability caused by headache, lower values were significantly associated with treatment resistant headache, lightheadedness, and abnormal levels of platelets on admission. Higher disability values were significantly associated with female sex, aggravation by physical activity, simultaneous photophobia and phonophobia, fever, expectoration, and abnormal leukocyte values on admission. The complete multivariate model is shown in Table 1. The interpretation of the coefficients is the same as for the intensity, noting that the score ranges between 0 and 100. In the case of the leukocyte levels, a value of the coefficient equal to 0.002 means that for each additional 1,000 leukocyte units (range 1,980–16,600 in this sample), the disability score is 2 points higher.

#### Migraine Symptoms

The multivariate logistic regression model for the migraine symptoms (criterion C from the ICHD-3) showed significantly higher odds associated with periocular pain, progressive worsening of the headache and criterion D of migraine. Lower odds were significantly associated with pressing pain and higher worst LDH levels. The complete model is shown in Table 2. In the logistic regression models, the Odds Ratio is employed to interpret the results. Values over 1 indicate a positive association, while values below 1 a negative association. For example, people with pressing pain presented 84% lower odds (Odds Ratio = 0.16) of developing migraine symptoms in comparison with people with no pressing pain. In the case of worst LDH values, the original coefficient from the logistic regression model was equal to −0.009 (Odds Ratio = 0.991); with an increase of 100 LDH units, the coefficient would be −0.9 and the Odds Ratio would be ~0.40, i.e., for each additional 100 LDH units (range 130–743 in this sample), the probability of developing migraine symptoms is around 60% lower.

**Table 2:** Multivariate logistic regression model of migraine characteristics (criteria C and D) in patients hospitalized because of COVID-19.

	**Odds ratio and 95% CI**	**Unadjusted *p*-value**	**Adjusted *p*-value (FDR)**
Migraine—Criterion C model			
Independent term	3.68 (0.48, 34.54)	0.22	0.22
**Pressing pain**	0.16 (0.03, 0.64)	0.013	0.037
**Migraine—Criterion D**	4.24 (1.29, 15.51)	0.021	0.047
**Periocular pain**	4.66 (1.45, 16.37)	0.012	0.037
**Progressive**	9.57 (1.89, 70.04)	0.013	0.037
**Worst LDH values**	0.991 (0.984, 0.997)	0.006	0.037
No response to analgesics	0.29 (0.05, 1.51)	0.15	0.16
Family history of headache	2.51 (0.84, 7.96)	0.10	0.13
Worst headache experienced in life	3.05 (0.97, 10.27)	0.061	0.085
Abnormal liver enzymes	3.45 (1.15, 11.48)	0.033	0.061
Prior history of neurological disorders	3.86 (1.05, 16.55)	0.052	0.081
Migraine—Criterion D model			
Independent term	0.13 (0.01, 1.45)	0.11	0.11
**Chest pain (COVID-19 symptom)**	0.12 (0.02, 0.47)	0.005	0.030
**Remitting headache**	0.16 (0.04, 0.64)	0.013	0.047
**Abnormal ferritin**	0.18 (0.03, 0.79)	0.028	0.047
**Disability caused by headache**	1.04 (1.01, 1.07)	0.005	0.030
**Diarrhea (COVID-19 symptom)**	3.42 (1.14, 11.39)	0.034	0.047
**Lymphopenia on admission**	3.95 (1.27, 13.92)	0.023	0.047
**Treatment resistant**	5.07 (1.34, 22.86)	0.024	0.047
**Prior history of diabetes**	6.64 (1.30, 42.47)	0.030	0.047
Worst headache experienced in life	2.98 (0.93, 10.49)	0.073	0.081
Treatment resistant (analgesics)	5.23 (0.98, 33.76)	0.063	0.077

*The bold terms show statiscally significant variables after the FDR correction (if a p-value is provided for the variable) or indicate a specific model or a variable with more than two categorical values*.

Lower odds in the model of criterion D of migraine were related to chest pain, remitting headache, and abnormal ferritin levels. Disability caused by headache, prior history of diabetes, diarrhea, abnormal levels of lymphocytes, and treatment-resistant headache were associated with higher odds of the fulfillment of criterion D of migraine. The complete model is shown in Table 2.

#### Topography (Frontal Region) and Quality of Pain

Higher odds of frontal pain were associated with female sex, bilateral diffuse pain, and abnormal interleukin-6 (IL-6) levels, and lower odds were related to cranial autonomic symptoms, after correcting for multiple comparisons. The complete frontal pain model is shown in Table 3.

**Table 3:** Multivariate logistic regression model of headache in the frontal area in patients hospitalized because of COVID-19.

	**Odds ratio and 95% CI**	**Unadjusted *p*-value**	**Adjusted *p*-value (FDR)**
Frontal pain model			
Independent term	0.12 (0.01, 1.05)	0.076	0.087
**Cranial autonomic symptoms**	0.049 (0.003, 0.63)	0.026	0.042
**Sex (Female vs. Male)**	20.59 (3.05, 286.23)	0.007	0.018
**Abnormal IL-6 levels**	36.93 (5.06, 605.14)	0.002	0.009
**Bilateral diffuse pain**	65.53 (8.24, 1247.40)	<0.001	0.006
Modified rankin scale at discharge	0.19 (0.04, 0.62)	0.011	0.021
Glomerular filtration rate on admission (interval)	0.22 (0.03, 1.16)	0.093	0.093
Pulsating	53.15 (2.44, 10459.82)	0.053	0.070
Pressing pain model			
Independent term	383.92 (6.53, 50195.26)	0.008	0.017
**Abnormal PCT on admission**	0.031 (0.001, 0.381)	0.011	0.021
**Modified rankin scale**	0.041 (0.003, 0.466)	0.014	0.021
**Clinophilia**	0.06 (0.01, 0.37)	0.007	0.017
**Headache when sleeping**	0.18 (0.04, 0.75)	0.023	0.027
**Headache intensity**	0.35 (0.17, 0.61)	0.001	0.005
**Prior history of diabetes**	88.00 (2.95, 9378.87)	0.024	0.027
**Pathologic chest X-Ray**	794.78 (29.10, 68399.71)	<0.001	0.005
No response to analgesics	0.31 (0.06, 1.34)	0.12	0.12

*The bold terms show statiscally significant variables after the FDR correction (if a p-value is provided for the variable) or indicate a specific model or a variable with more than two categorical values*.

Lower odds of pressing pain were associated with headache intensity, headache when sleeping (and with consequent waking-up), modified Rankin scale, abnormal PCT levels and clinophilia. Higher odds of pressing pain with diabetes and pathologic chest X-Ray were identified. The complete model is shown in Table 3. Anyway, the extremely high and low values represented in Table 3 indicate overfitting, and Odds Ratios should be interpreted with high caution.

## Discussion

**Figure 5:**
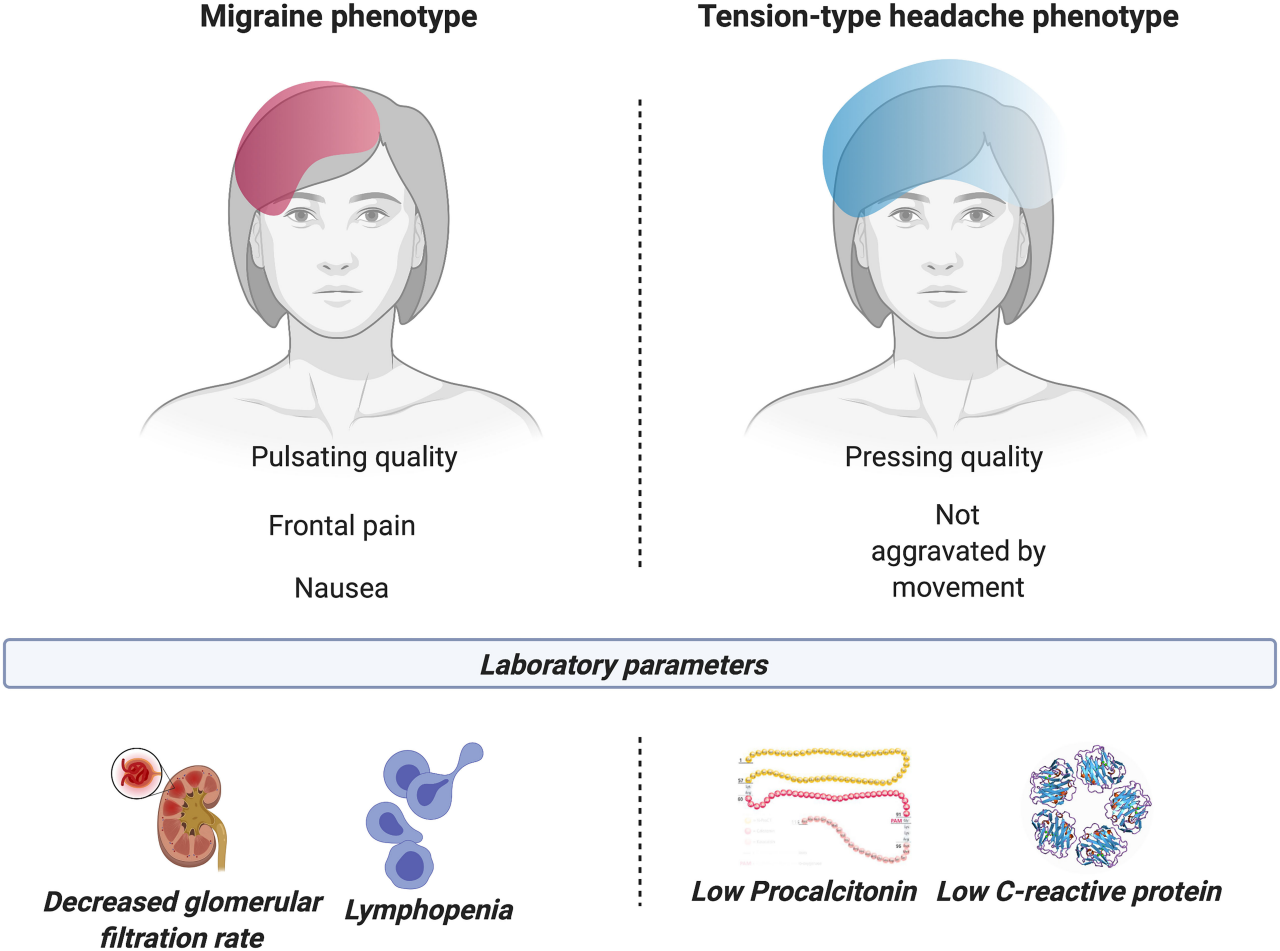
Summary of the laboratory tests (worst levels and values on admission) in association with headache phenotypes. Symptoms of migraine and TTH were linked to laboratory biomarkers of COVID-19 status.

The results from this study showed that intensity and disability caused by headache attributed to COVID-19 were associated with the disease state and certain symptoms, mainly fever, and two different phenotypes. The first phenotype is the headache with migraine features (specifically pulsating quality and nausea during headache), related to high intensity and disability caused by headache itself, to hematologic and inflammatory potential biomarkers of COVID-19 severity such as thrombocytopenia, lymphopenia, and hyperferritinemia. The second phenotype presents TTH characteristics (particularly pressing quality, not aggravated by movement and mild or moderate pain intensity) and is linked to lower PCT and CRP levels, both potential biomarkers of COVID-19 severity when high levels are present. A last possible phenotype would be a COVID-19 specific phenotype, associated with lymphopenia and high levels of PCT and CRP, and characterized by bilateral (diffuse) frontally localized, pressing, and intense pain, and hypersensitivity to stimuli. A summary of the results is shown in Figure 5.

PCA was employed to overcome the limitations of regression. PCA allows to analyze simultaneously a set of variables and detect specific patterns depending on predictor variables. A principal component is influenced by the effect of the variables included in the analysis, each one with a specific weight per component. In this study, for example, the PCA showed that CRP and PCT levels followed simultaneous changes in relationship with diverse headache features. The identification of concurrent changes in some variables is a key advantage of PCA in comparison with regression analysis, which analyzes a unique dependent variable per model.

Considering the relationship between the primary headache features, migraine and TTH in this case, and a secondary headache attributed to COVID-19, a possible explanatory mechanism would be related to pre-existing primary migraine and TTH. Schankin and Straube observed that sometimes secondary headaches are strongly related to pre-existing primary headaches (14). In our study, we found no statistically significant associations between phenotypes related to migraine and TTH characteristics, such as higher/lower intensity of pain and pulsating or pressing quality, and previous history of migraine and TTH, respectively. However, the number of patients with prior history of primary headache was relatively small in our sample, including 18 and 30 patients with previous migraine and TTH, respectively. Further studies with higher sample size should elucidate whether diverse pre-existing primary headaches, mainly migraine and prior family history of migraine, influence the different manifestations of headache attributed to COVID-19.

Concerning the identification of headache phenotypes, higher values of intensity of headache and self-rated disability were associated with symptoms related to migraine and female sex, which is substantially more common in migraine. These results suggest that phenotypic features of migraine should be monitored in COVID-19 patients in relation to the course of the disease, considering that it may affect patients' quality of life.

Furthermore, higher disability values were associated with COVID-19 symptoms and results from laboratory tests. Patients with fever presented the highest disability scores in our sample. Headache intensity has been previously associated with fever in COVID-19 (3) and non-cephalic infections, and headache has also been hypothesized as a secondary effect of fever (15). In contrast, lightheadedness was associated with lower disability according to our results (Table 1). However, people with lightheadedness and fever exhibited higher disability than patients with none of them, considering that almost all patients with lightheadedness also presented fever. This result is in line with reported higher dizziness in patients with migraine compared to control subjects, although the presence of migraine did not increase the risk of dizziness (16).

Regarding the relationship between disability caused by headache and laboratory tests, leukocytosis on admission was associated with higher disability, and abnormal (very high or low) platelet levels on admission with lower disability. On the one hand, thrombocytopenia has been observed in patients with headache characteristics related to migraine, with attacks occurring regularly during periods of thrombocytopenia and relieve of migraine symptoms associated with its normalization (17–19). Thrombocytopenia has also been related to increased risk of severe COVID-19 (20, 21). On the other hand, platelet-leukocyte aggregates count has been found to be higher in patients with migraine during the interictal period in comparison with control subjects (22), with a possible link to the release of IL-6 and cytokine tumoral necrosis factor-α (19). Therefore, our results suggest that higher disability caused by headache may be related to a phenotype with migraine features, with possible platelet and platelet-leukocyte complex pathophysiological underlying mechanisms.

Following laboratory tests results, we found that abnormal (higher) levels of IL-6 were associated with pain in the frontal region, the most common region with pain in our sample. IL-6 has been found to be increased in patients with TTH and migraine during headache compared to controls (23, 24). These increased levels in both primary headaches may explain the reason of the lack of association with specific migraine or TTH features. With regard to the pathophysiological mechanism of IL-6, a sensitization of the dural afferents has been suggested to contribute to migraine pathophysiology in association with IL-6 in the meninges (24). Moreover, IL-6 blood measurement has been pointed out as a potential biomarker of COVID-19 severity (25). We also identified that other laboratory results, such as lymphopenia, were also associated with migraine features. This relationship between frontal pain in headache in patients with COVID-19 and headache with migraine characteristics may reflect the connection between disability, or even severity of COVID-19, and headache in some patients with COVID-19. IL-6 may play an important role in the generation of headache attributed to COVID-19. In rat models, calcitonin gene-related peptide, strongly related to migraine headache (26), was shown to be released in heat conditions in association with IL-6 (27).

With reference to the lymphopenia in the phenotype with migraine features, a lower lymphocyte count value has been found in patients with severe COVID-19 (21). Furthermore, we found that abnormal (higher) ferritin levels were associated with criterion D for migraine from the ICHD-3. Higher serum ferritin levels have been found in patients with severe COVID-19 (21). These results may imply a relationship between COVID-19 state, laboratory biomarkers and headache, with a phenotype with migraine characteristics related possibly to severe COVID-19.

In accordance with the relationship between lymphocyte and ferritin values, compared to the phenotype with migraine characteristics, lower CRP and PCT values (on admission and worst levels) were found in patients with a phenotype with TTH features. Considering that TTH features used in this study oppose migraine characteristics (e.g., headache not aggravated by physical activity in TTH, and aggravated by activity in migraine), these results are in line with previous COVID-19 and migraine studies. Severe COVID-19 has been found in patients with high CRP and PCT levels (21), and high CRP levels have been identified in patients with migraine with relative high frequency (seven or more days with headache per month) and chronic migraine (28), and migraine with aura (28, 29). High CRP levels have been identified to be correlated with a cytokine storm, showing high CRP levels in patients with severe COVID-19 (30). Increased circulating pro-inflammatory cytokines have been suggested to be related to headache attributed to COVID-19, triggering perivascular trigeminal nerve endings (4). Additionally, we measured D-dimer levels, with high levels found previously in patients with COVID-19 (21), but we identified no clear pattern related to D-dimer.

In this study, frequent symptoms of headache were associated with biomarkers of endothelium damage, such as high PCT and CRP levels. PCT and CRP have been proposed as agents that impair endothelial cell function (31, 32), and the endothelium may be a key target in COVID-19 (33). Considering that biomarkers of endothelial damage have been found in patients with migraine (34), the possible damage of endothelium related to COVID-19 may suggest a possible relationship between severity of COVID-19 and headache, specific of COVID-19 or related to migraine symptomatology. Interestingly, a significant positive association was found between patients with prior history of diabetes and migraine symptoms, i.e., prior history diabetes was related to higher odds of presenting headache with migraine features. Considering the diverse mechanisms of endothelial dysfunction in diabetes (35), the endothelium damage in COVID-19 and migraine may explain that patients with diabetes suffered with higher odds a headache with migraine characteristics. This endothelial damage would cause headache, but perhaps it would not generate further complication related to an extremely high severe COVID-19 state.

Higher odds of presenting headache with migraine features were also associated with progressive pain. This result may imply that the phenotype with migraine characteristics would not appear at the beginning of the COVID-19 course, but in a later stage, related possibly to a worse COVID-19 state. Other factors associated with a headache with migraine characteristics were persistent and treatment resistant headache. These previous characteristics, together with frequent features observed in this sample such as intense and frontally localized pain, have been reported in drug-induced aseptic meningitis, which also presents migraine symptoms occasionally (36). Viral meningitis has been proposed as a neuropathological mechanism of COVID-19 (37). Hence, the frequent symptoms found in this sample suggest that headache related to COVID-19 may be linked to meningeal irritation, in line with the previously commented IL-6 pathophysiological mechanisms related to calcitonin gene-related peptide release.

The presence of the most common headache symptoms in this sample (frontal or bilateral diffuse pain, intense pain, pressing pain, and hypersensitivity to stimuli) was related to the laboratory biomarkers of severe COVID-19 state, i.e., lymphopenia and high values of CRP and PCT. These features combine characteristics from both migraine and TTH, which may explain that some subjects simultaneously fulfilled criteria C for migraine and TTH from the ICHD-3. Furthermore, these features may define a third phenotype which could be “COVID-19 specific,” which would be more intense than a headache with TTH characteristics, but not than a phenotype with migraine characteristics. The existence of diverse phenotypes is in line with heterogeneity and several patterns of headache during COVID-19 infection reported in healthcare workers (38). A possible factor related to the commented “COVID-19 specific” phenotype may be anosmia, one of the most characteristic symptoms of COVID-19 (39). Talavera et al. (40) found that COVID-19 patients with anosmia, compared to those without anosmia, presented a higher prevalence of headache and lower severity and mortality rate, and also higher values of lymphocyte count, increased glomerular filtration rate, and lower CRP levels. These values would be in line with our results, which showed higher glomerular filtration rate and lymphopenia in patients with migraine features, associated with a more severe course in this study, and lower CRP levels, linked to the phenotype with TTH features. Thus, anosmia may be related to a headache phenotype characteristic of COVID-19, possibly with no association with a severe course of the disease. The possible intermediate headache intensity and disability between migraine and TTH of the suggested “COVID-19 specific” phenotype may explain the lack of statistically significant results of anosmia in this study. Future studies should analyze with more detail the relationship between anosmia and headache during COVID-19.

Some limitations are worth mentioning in this study. Only hospitalized patients were included in the study because they were recruited at the beginning of the COVID-19 crisis, which implied that RT-PCR diagnostic test were used mainly in patients who needed hospital admission. No patients with extremely severe condition, including death and patients who were not able to take part in the interview, were included in the sample, which limits the results related to COVID-19 severity. Sample size was another important limitation. Although sample size was not excessively small, some adjustments were necessary to avoid extreme overfitting effects in the multivariate GLM. Also, more sophisticated clustering methods than PCA, such as k-means, were not possible because of this. The phenotype with TTH features that we associated with lower intense headache was obtained in patients that had a relative high pain intensity and needed admission, and we were not able to elucidate whether mild state (or mild headache intensity) patients with COVID-19 presented the same phenotype or a different one. The cross-sectional nature of the study was another factor that made it impossible to assess whether a single patient could present different headache phenotypes depending on the course of the disease. In relation with the longitudinal course of headache during COVID-19, there was no headache diary available for each patient and the analysis was limited to the inpatient stay, with no chance to assess the evolution of the symptoms. Further studies should try to avoid these limitations using a validated structured interview and a headache diary for a better evaluation of the course of headache.

In conclusion, headache attributed to COVID-19 can be manifested in diverse phenotypes associated with migraine and TTH characteristics. The phenotype associated with migraine symptoms was related to a worse clinical course of COVID-19, including a relationship with hematologic and inflammatory markers of the disease, and it was also linked to higher intensity and disability caused by headache. Headache should be considered as an important symptom in the clinical course of COVID-19, especially when manifested with migraine characteristics, taking into account that it is a common and disabling symptom of COVID-19. Future studies should assess the longitudinal evolution of headache in patients with COVID-19 to characterize accurately the different headache phenotypes and their relationship with the clinical course of the disease.

## Data Availability Statement

The raw data supporting the conclusions of this article will be made available by the authors, without undue reservation.

## Ethics Statement

The studies involving human participants were reviewed and approved by Valladolid East Ethics review board. The patients/participants provided their written informed consent to participate in this study.

## Author Contributions

ÁP-G, ÁLG, and DG-A designed and conceptualized the study. JT and DG-A collected the data. ÁP-G and DG-A analyzed and interpreted the data and drafted the manuscript. RdL-G, ÁLG, and JP-E revised the manuscript for intellectual content. All authors agreed and approved the manuscript.

## Conflict of Interest

The authors declare that the research was conducted in the absence of any commercial or financial relationships that could be construed as a potential conflict of interest.

## Abbreviations

COVID-19: coronavirus disease 2019
CRP: C-reactive protein
FDR: false discovery rate
GLM: generalized linear model
ICHD-3: The International Classification of Headache Disorders (3rd edition)
IL-6: interleukin-6
LDH: lactate dehydrogenase
PCA: principal component analysis
CT: procalcitonin
RT-PCR: real-time reverse-transcriptase-polymerase-chain-reaction
TTH: tension-type headache.
