# Supplementary material for: Deep Phenotyping of Headache in Hospitalized COVID-19 Patients via Principal Component Analysis
Source: Front Neurol. 2020 Dec 17;11:583870. doi: 10.3389/fneur.2020.583870 (PMC7773780; doi:10.3389/fneur.2020.583870)
Supplement: Supplementary file 1 [file Data_Sheet_1.PDF]

Supplementary Material

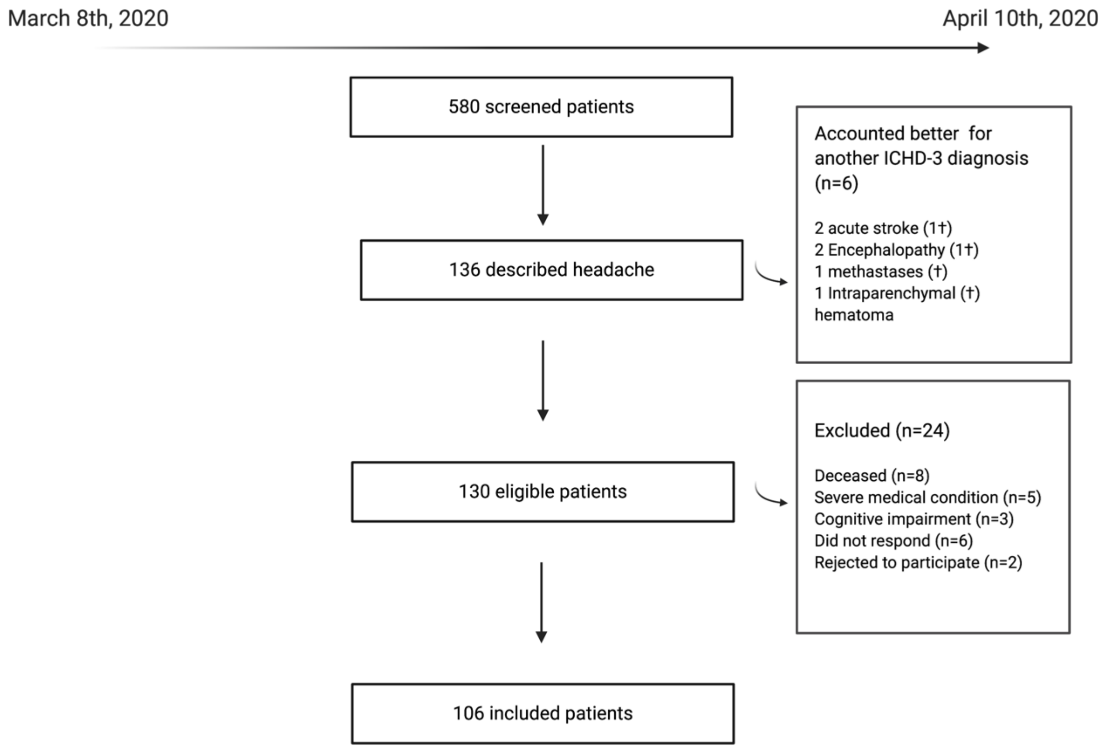

Supplementary Figure 1. Flow diagram of the patients included in the study.

Supplementary Table 1. Laboratory tests and prior medical history analyzed in the sample.

Variable	Levels	Unit	Reference value
Arterial hypertension	Yes/no (two prior determinations)	mmHg	Higher than 140/90
Cancer (history)	Yes/no		
Cardiovascular disease	Yes/no		
Chronic pulmonary disease	Yes/no		
Creatine phosphokinase	Continuous and abnormal (admission and worst; yes/no)	U/L	20-170
C-reactive protein	- Continuous and abnormal (admission and worst; yes/no)	mg/L	1-5

	- Days after admission with worst levels<br>- Days with worst levels		
D-dimer	Continuous and abnormal (admission and worst; yes/no)	ng/dL	< 500
Diabetes	Yes/no	- mg or mg/dl<br>- mg/dl<br>- %	- > 200 (oral or blood glucose with symptoms)<br>- > 126 (two tests)<br>- HbA1c > 6.5
Ferritin	Continuous and abnormal (worst; yes/no)	ng/dL	15-150
Glomerular filtration rate	- Continuous and abnormal (admission and worst; yes/no)<br>- Abnormal interval (first and admission)	$ml/min/1.73m^2$	> 90
Hemoglobin	Continuous and abnormal (admission and worst; yes/no)	g/dL	12-16
Hepatic disease	Yes/no		
Immunocompromised state	Yes/no		
Interleukin-6	Continuous and abnormal (worst; yes/no)	pg/mL	< 5.9
International Normalized Ratio	Continuous and abnormal (admission and worst; yes/no)		0-8-1.3
Lactate dehydrogenase	- Continuous and abnormal (admission and worst; yes/no)	U/L	135-250
Leukocyte	- Continuous and abnormal (admission and worst; yes/no)	cell count · 10^9/L	4-10
Lymphocyte	- Continuous and abnormal (admission and worst; yes/no)<br>- Days with worst count<br>- Days after admission with worst count	count · 10^9/L	0.9-5.2

Neurological disorders (history)	Yes/no		
Platelet	- Continuous and abnormal (admission and worst; yes/no)	count · 10^9/L	150-400
Procalcitonin	- Continuous and abnormal (admission and worst; yes/no)	ng/mL	< 5
Smoking habit (present or discontinued in the last six months)	Yes/no		

Supplementary Table 2. Significant univariate GLM of the headache intensity and self-reported disability in patients hospitalized because of COVID-19.

	Coefficient and 95% CI	P-value	Missing
Intensity of headache			
**Treatment resistant**	-1.36 [-2.00, -0.71]	< 0.001	0
**Sex (Female vs. Male)**	1.17 [0.48, 1.86]	0.001	0
Treatment resistant (analgesics)	1.21 [0.24, 2.18]	0.017	0
Pressing pain	-1.60 [-2.35, -0.86]	< 0.001	0
Pulsating pain	0.88 [0.05, 1.71]	0.040	0
**Aggravation by physical activity**	1.25 [0.55, 1.94]	< 0.001	0
Migraine – Criterion C	1.28 [0.62, 1.93]	< 0.001	0
Migraine – Criterion D	0.85 [0.15, 1.55]	0.019	0
**Phonophobia and photophobia**	1.01 [0.26, 1.75]	0.009	0
**TTH – Criterion C**	-1.21 [-2.16, -0.27]	0.013	0
TTH – Criterion D	-0.85 [-1.55, -0.15]	0.019	0
Smoking (previous or current)	-1.42 [-2.49, -0.36]	0.010	0
CPK values on admission	-0.004 [-0.008, - 0.000]	0.046	65
Days with worst lymphocyte count	-0.13 [-0.21, -0.04]	0.005	0
Worst ferritin values	-4.27 [-8.26, -0.27] $\cdot 10^{-4}$	0.039	1
**Abnormal platelet levels on admission**	-1.20 [-2.17, -0.22]	0.018	0
Abnormal worst platelet levels	-0.77 [-1.48, -0.05]	0.038	0
Similar to previous headache (0-100)	-0.02 [-0.04, -0.01]	0.001	45
**Headache as first COVID-19 symptom**	1.13 [0.37, 1.89]	0.004	0
Persistent headache	0.70 [0.02, 1.39]	0.048	0
Headache when sleeping	1.09 [0.19, 2.00]	0.020	0
Clinophilia	0.86 [0.17, 1.55]	0.016	0
Maneuvers as trigger	0.81 [0.11, 1.51]	0.026	0
**Aggravation by head move**	1.10 [0.37, 1.82]	0.004	0
**Quality of headache**			0
Pulsating vs. Pressing	1.49 [0.41, 2.56]	0.008	
Stabbing vs. pressing	1.40 [0.22, 2.57]	0.022	
Self-reported disability			
**Treatment resistant**	-21.60 [-31.31, -11.90]	< 0.001	0
**Sex (Female vs. Male)**	14.93 [4.31, 25.55]	0.007	0
**Age (50 or more vs. less than 50)**	-13.39 [-24.81, -1.96]	0.024	0
Treatment resistant (analgesics)	17.78 [3.03, 32.53]	0.020	0
Periocular pain	13.54 [2.34, 24.73]	0.020	0
**Neurological symptoms of headache**	16.01 [2.30, 29.72]	0.024	0
**Red flag (Past Medical History)**	-13.70 [-25.68, -1.72]	0.027	0

Pressing pain	-19.31 [-31.00, -7.62]	0.002	0
Diffuse pain	14.75 [3.89, 25.61]	0.009	0
**Bilateral diffuse pain**	11.47 [1.10, 21.84]	0.032	0
**Aggravation by physical activity**	20.68 [10.26, 20.67]	< 0.001	0
Migraine – Criterion C	15.85 [5.74, 25.97]	0.003	0
Migraine – Criterion D	16.48 [6.07, 26.90]	0.003	0
**Phonophobia and photophobia**	21.21 [10.35, 32.07]	< 0.001	0
TTH – Criterion C	-20.33 [-34.55, -6.12]	0.006	0
TTH – Criterion D	-16.48 [-26.90, -6.07]	0.003	0
**Smoking (previous or current)**	-24.70 [-40.66, -8.74]	0.003	0
**Fever**	22.33 [5.56, 39.09]	0.010	0
**Lightheadedness**	-15.61 [-30.45, -0.77]	0.004	0
Days with worst lymphocyte count	-1.95 [-3.25, -0.64]	0.001	0
**Days from admission to worst lymphocyte count**	-1.05 [-1.84, -0.26]	0.011	0
Abnormal worst platelet levels	-11.26 [-22.12, -0.40]	0.045	0
Similar to previous headache (0-100)	-0.36 [-0.57, -0.15]	0.001	45
Hours with headache	3.25 [0.60, 5.90]	0.020	47
Hypersensitivity to stimuli	10.98 [0.54, 21.41]	0.042	0
Clinophilia	22.90 [13.18, 32.63]	< 0.001	0
Maneuvers as trigger	14.88 [4.37, 25.38]	0.007	0
Aggravation by head move	22.87 [12.35, 33.38]	< 0.001	0
Aggravation by eyes move	14.50 [1.30, 27.70]	0.034	0
**Quality of headache**			0
Pulsating vs. pressing	20.51 [3.55, 37.37]	0.020	

Variables in bold were included in the multivariate model. CPK = creatine phosphokinase; TTH = tension-type headache.

Supplementary Table 3. Principal Component Analysis results of the intensity and disability.

	PC1	PC2	PC3	PC4
Intensity and disability + Abnormal platelet levels				
**Relative contribution (%)**				
Intensity	33.1	16.3	0.4	50.3
Disability	31.5	19.2	0.4	48.8
**Coefficient**				
Intensity	0.317	0.222	-0.034	0.391
Disability	0.020	0.016	0.002	-0.025
Intensity and disability + COVID-19 symptoms				
**Relative contribution (%)**				
Intensity	41.7	0.4	11.9	46.0
Disability	44.9	0.3	2.9	51.8
**Coefficient**				
Intensity	0.356	0.036	0.190	-0.373
Disability	0.024	-0.002	0.006	0.026
Intensity and disability + Migraine symptoms				
**Relative contribution (%)**				
Intensity	27.4	4.6	0.3	3.3
Disability	27.5	3.1	0.9	0.8
**Coefficient**				
Intensity	0.288	-0.118	-0.030	-0.100
Disability	0.019	-0.006	-0.003	-0.003
Intensity and disability + Criteria C-D for migraine, C for TTH				
**Relative contribution (%)**				
Intensity	29.5	6.4	14.4	1.9
Disability	29.6	9.0	11.2	1.5
**Coefficient**				
Intensity	0.299	0.140	0.209	-0.076
Disability	0.020	0.011	0.012	0.005
Intensity and disability + Quality of pain				
**Relative contribution (%)**				
Intensity	29.4	6.1	0.3	6.8
Disability	24.6	11.0	0.1	21.6
**Coefficient**				
Intensity	0.299	-0.136	0.028	0.143

Disability	0.018	-0.012	0.001	0.017
Intensity and disability + Topography-laterality				
**Relative contribution (%)**				
Intensity	28.5	20.7	0.8	2.0
Disability	34.3	11.2	1.9	1.4
**Coefficient**				
Intensity	0.294	0.251	-0.050	-0.079
Disability	0.021	0.012	-0.005	-0.004
Intensity and disability + Most frequent symptoms				
**Relative contribution (%)**				
Intensity	34.3	0.09	1.35	0.69
Disability	32.5	2.29	0.04	0.73
**Coefficient**				
Intensity	0.323	0.017	0.064	-0.046
Disability	0.021	0.006	-0.001	-0.003

PC = Principal Component; TTH = tension-type headache. Categorical variables were abnormal platelet levels (first and worst levels, categorical variables), COVID-19 symptoms (fever and lightheadedness), migraine symptoms (unilateral, pulsating, and intense pain, aggravation by physical activity, nausea, and simultaneous phonophobia and photophobia), criteria C-D for migraine, and criterion C for tension-type headache, from the ICHD-3, quality of headache (pressing, pulsating, and stabbing pain), and topography-laterality of headache (frontal, temporal, periocular, and bilateral diffuse pain).

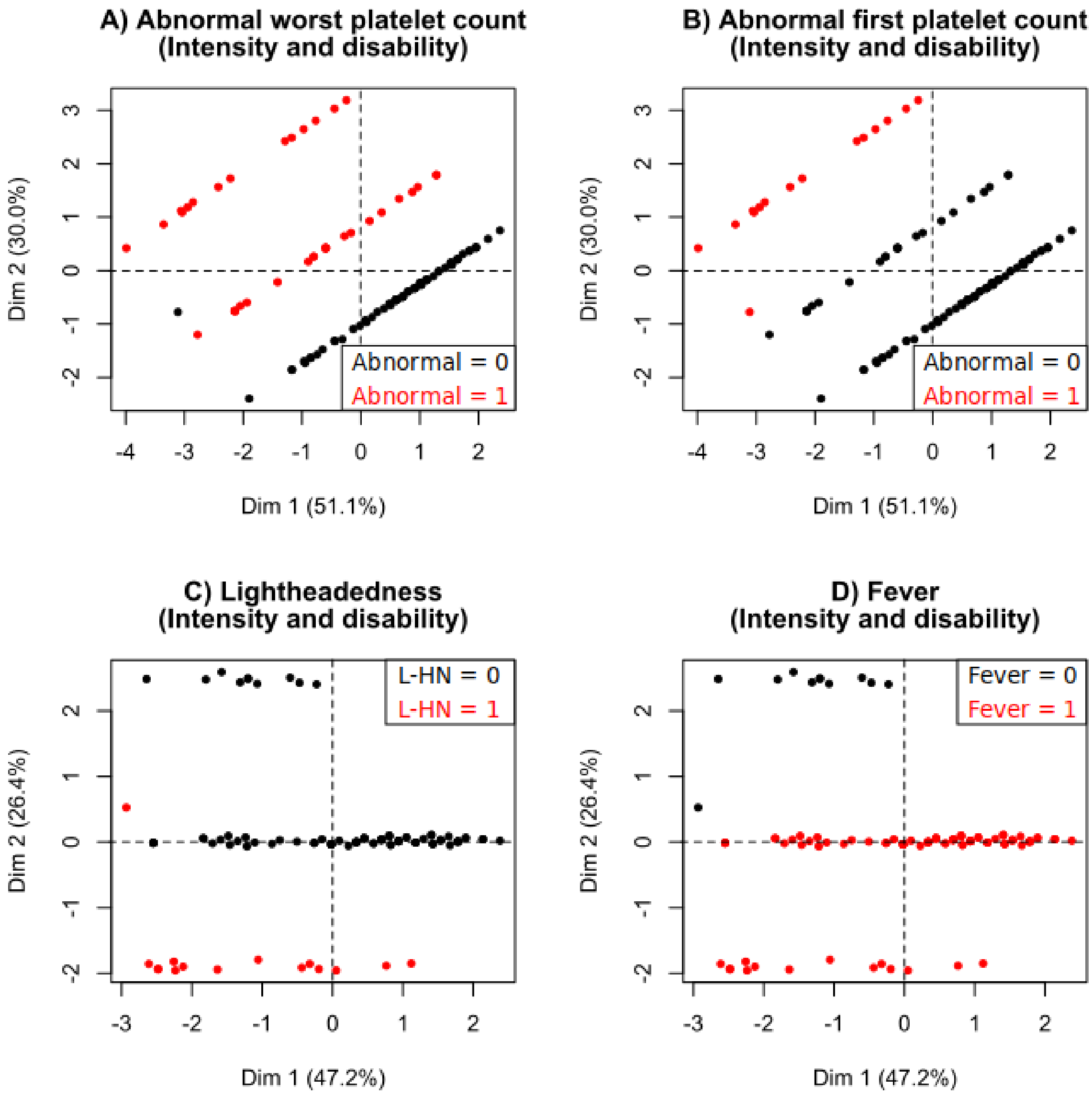

Supplementary Figure 2. Mix PCA of headache intensity and disability caused by headache, and abnormal laboratory tests results (platelets) and COVID-19 symptoms (Lightheadedness and Fever). X- and Y -axis contain the values of the first and second principal components, respectively. 0 = no symptom or abnormal level; 1 = present symptom or abnormal level.

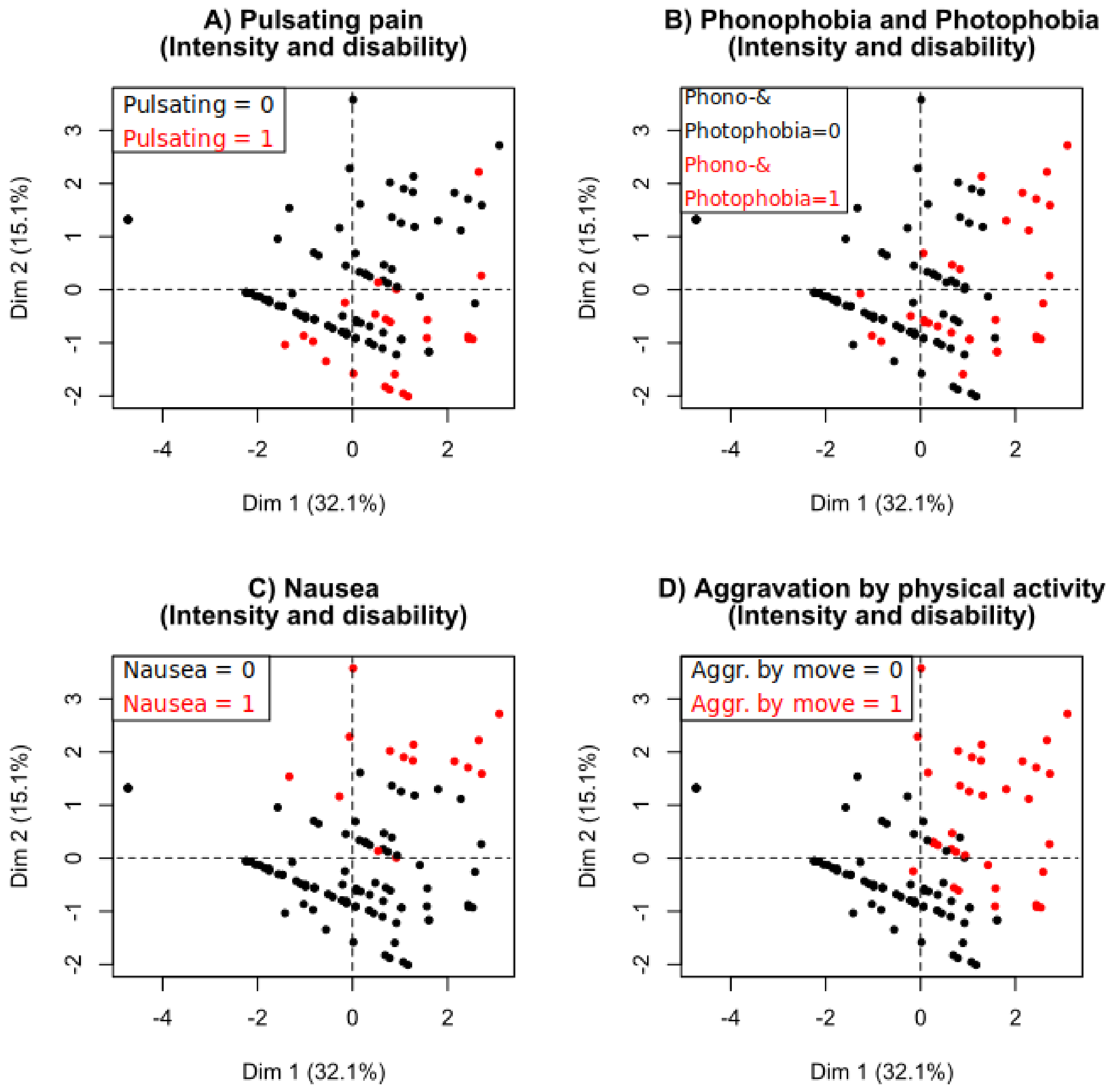

Supplementary Figure 3. Mix PCA of headache intensity and disability caused by headache, and diverse migraine symptoms. X- and Y-axis contain the values of the first and second principal components, respectively. 0 = no symptom; 1 = present symptom.

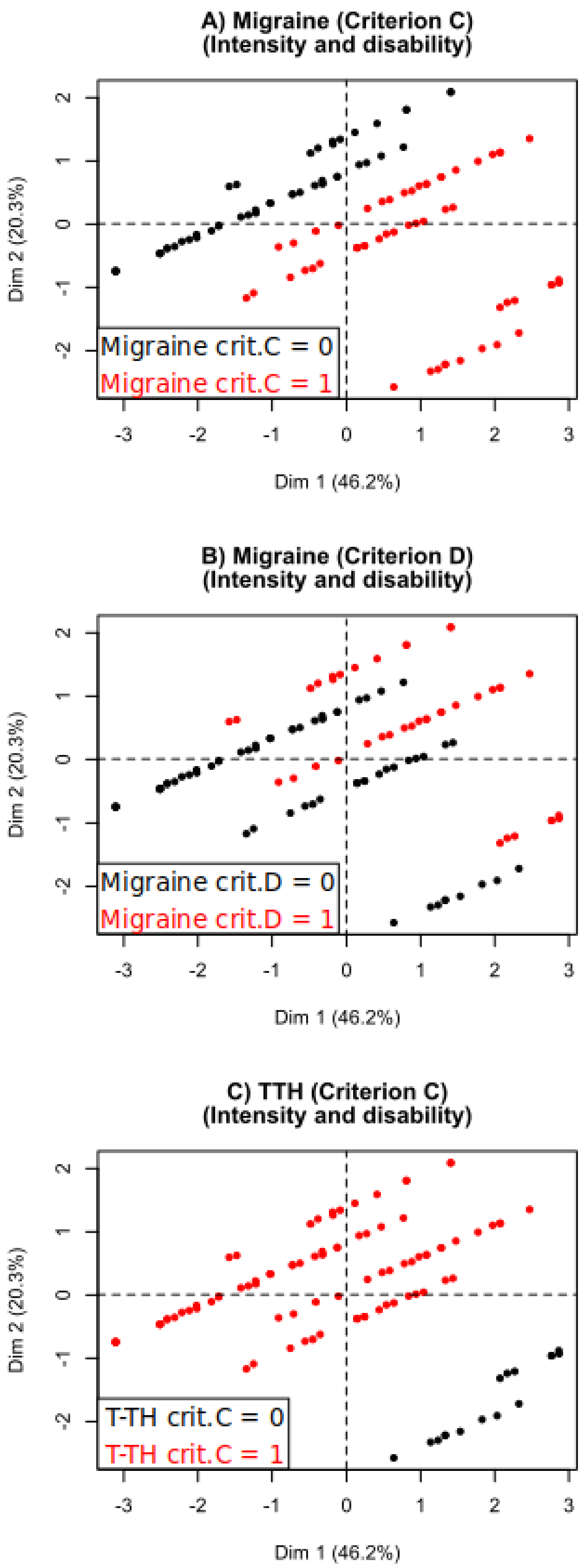
A) Migraine (Criterion C)
(Intensity and disability)
Migraine crit.C = 0
Migraine crit.C = 1
B) Migraine (Criterion D)
(Intensity and disability)
Migraine crit.D = 0
Migraine crit.D = 1
C) TTH (Criterion C)
(Intensity and disability)
T-TH crit.C = 0
T-TH crit.C = 1
Dim 1 (46.2%)
Dim 2 (20.3%)
-3
-2
-1
0
1
2
3

Supplementary Figure 4. Mix PCA of headache intensity and disability caused by headache, and criteria C and D for migraine, and C for TTH, from the ICHD-3. X- and Y-axis contain the values of the first and second principal components, respectively. 0 = does not meet criterion; 1 = meets criterion.

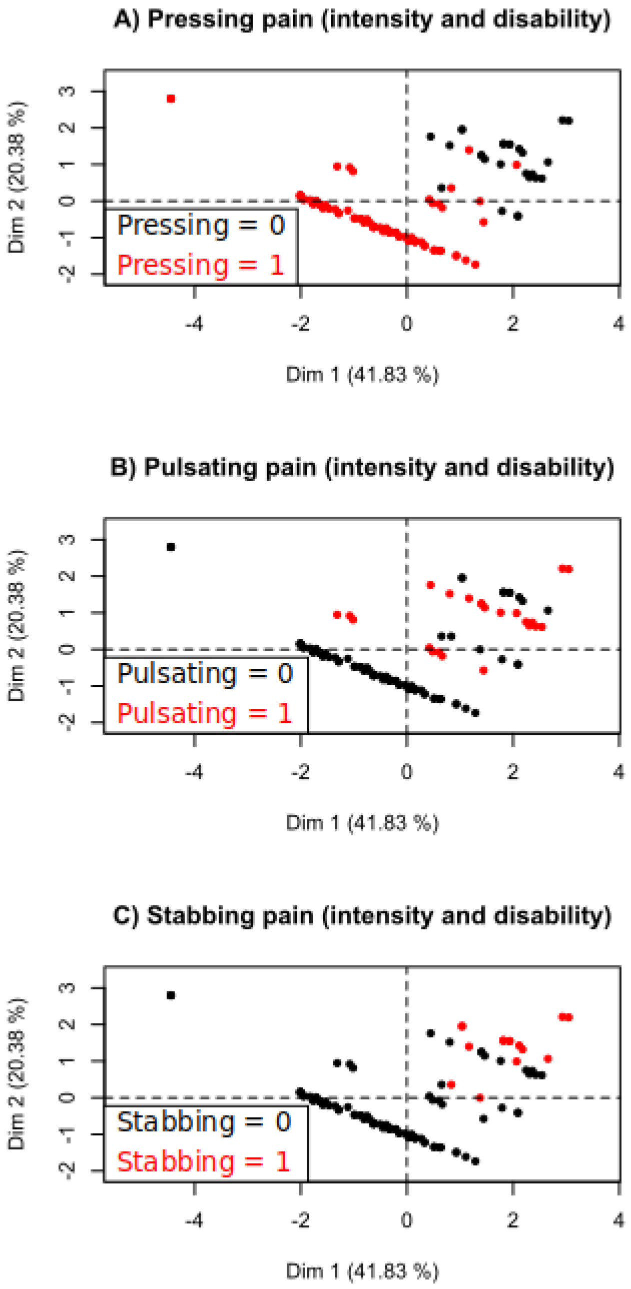

Supplementary Figure 5. Mix PCA of headache intensity and disability caused by headache, and quality of pain. X- and Y-axis contain the values of the first and second principal components, respectively. 0 = no quality; 1 = present quality.

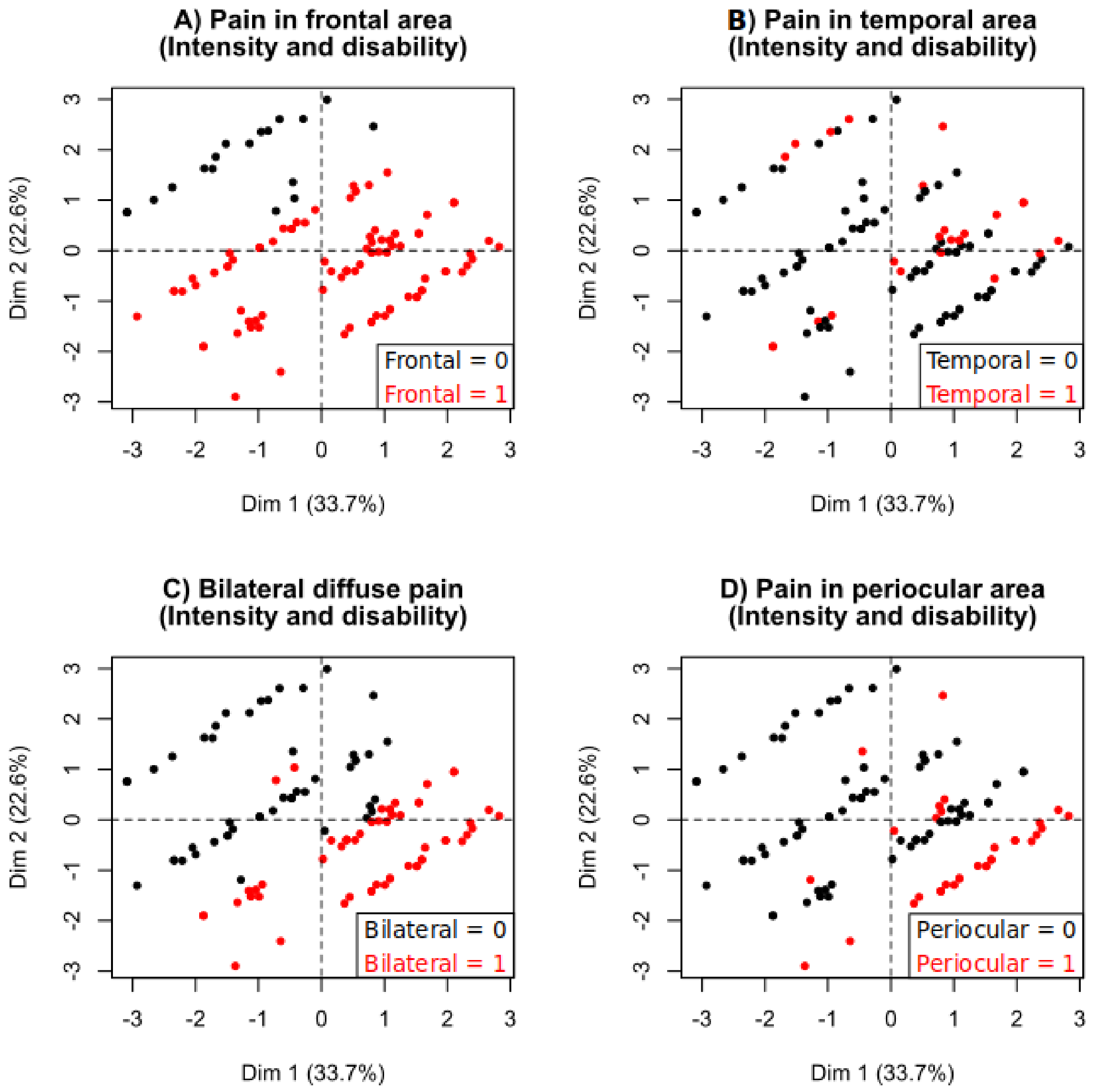

Supplementary Figure 6 Mix PCA of headache intensity and disability caused by headache, and location and topography of pain. X- and Y-axis contain the values of the first and second principal components, respectively. 0 = absent location/topology; 1 = present location/topography.

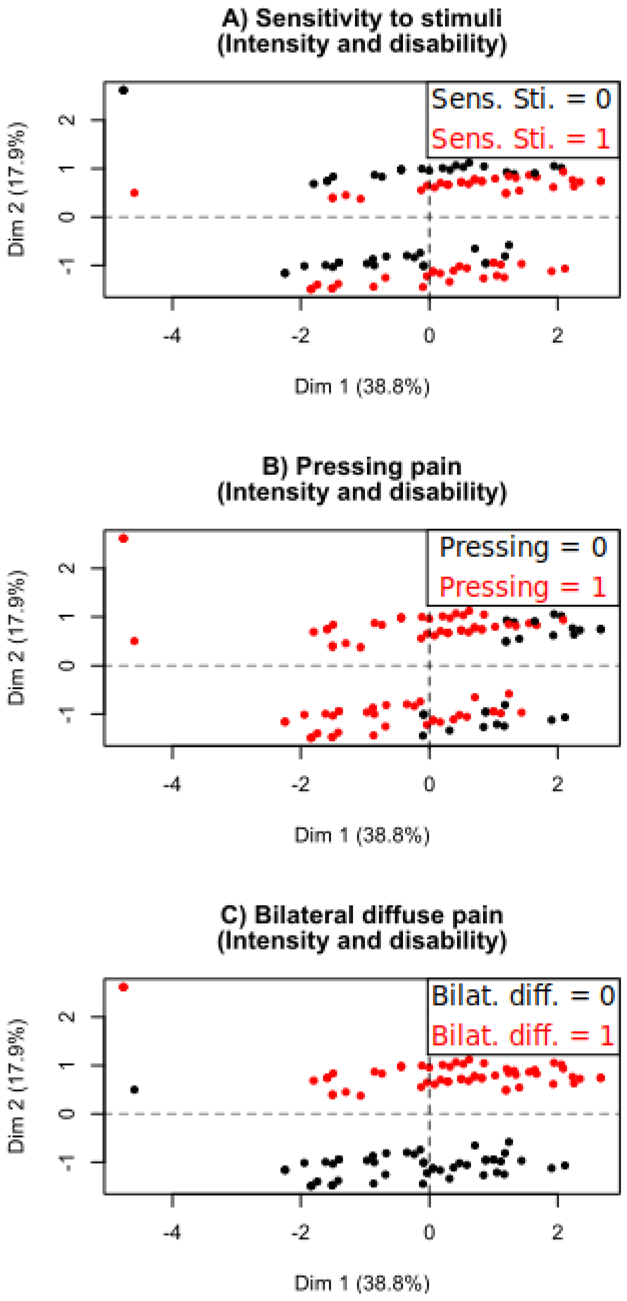

Supplementary Figure 7. Mix PCA of headache intensity and disability caused by headache, and most frequent characteristics found in the sample. X- and Y-axis contain the values of the first and second principal components, respectively. 0 = absent characteristic; 1 = present characteristic.

Supplementary Table 4. Significant univariate logistic regression models of migraine characteristics (criterion C and D of the ICHD-3) in patients hospitalized because of COVID-19.

	Odds Ratio and 95% CI	P-value	Missing
Criterion C of migraine – ICHD-3			
Headache intensity	1.54 [1.22, 2.00]	< 0.001	0
**Disability caused by headache**	1.023 [1.008, 1.039]	0.004	0
**Treatment resistant**	0.40 [0.18, 0.86]	0.021	0
**Periocular pain**	5.24 [2.10, 14.56]	< 0.001	0
**Pressing pain**	0.23 [0.08, 0.61]	0.005	0
Diffuse pain	2.94 [1.28, 7.02]	0.012	0
**Migraine – Criterion D**	2.82 [1.26, 6.56]	0.014	0
TTH – Criterion D	0.35 [0.15, 0.80]	0.014	0
No nausea	0.13 [0.02, 0.51]	0.010	0
LDH values on admission	0.994 [0.989, 0.999]	0.014	2
Glomerular filtration rate on admission	0.96 [0.92, 0.99]	0.026	4
**Worst LDH values**	0.995 [0.990, 0.999]	0.018	1
Worst glomerular filtration rate	0.97 [0.95, 0.99]	0.047	3
**Family history of headache**	2.37 [1.06, 5.44]	0.037	0
Days with headache	1.08 [1.01, 1.17]	0.042	20
Duration of headache attacks (hours)	1.34 [1.04, 1.80]	0.036	47
Headache when sleeping	4.01 [1.32, 15.03]	0.022	0
**Worst headache experienced in life**	3.37 [1.49, 7.99]	0.004	0
Aggravation by eyes move	11.92 [3.18, 77.97]	0.001	0
Vegetative symptoms	7.58 [1.95, 50.28]	0.010	0
Criterion D of migraine – ICHD-3			
Headache intensity	1.32 [1.05, 1.69]	0.022	0
**Disability caused by headache**	1.02 [1.01, 1.04]	0.004	0
**Treatment resistant (analgesics)**	5.88 [1.84, 22.68]	0.005	0
Periocular pain	2.51 [1.08, 5.96]	0.034	0
Previous headache	2.33 [1.03, 5.49]	0.046	0
Diffuse pain	2.77 [1.14, 7.29]	0.029	0
Worsens with move	3.13 [1.36, 7.34]	0.008	0
Migraine – Criterion C	2.82 [1.26, 6.56]	0.014	0
Mild to moderate pain	0.37 [0.15, 0.88]	0.029	0
**Prior history of diabetes**	6.10 [1.69, 28.96]	0.010	0
**Chest pain (COVID-19 symptom)**	0.27 [0.08, 0.73]	0.015	0
Abnormal lymphocyte count on admission	3.05 [1.17, 8.25]	0.024	0
Hemoglobin normal levels on admission	2.41 [1.06, 5.56]	0.036	0
Similar to headache attributed to systemic viral infection	0.28 [0.08, 0.88]	0.034	55

Persistent COVID-19	3.05 [1.17, 8.25]	0.024	0
**Remitting headache**	0.25 [0.08, 0.67]	0.007	0
**Worst headache experienced in life**	2.30 [1.03, 5.24]	0.044	0
Clinophilia	2.80 [1.23, 6.73]	0.017	0
Maneuvers as trigger	3.90 [1.71, 9.17]	0.001	0
Aggravation by head move	2.78 [1.20, 6.59]	0.018	0
Aggravation by eyes move	3.11 [1.16, 8.77]	0.027	0

Variables in bold were included in the multivariate model. LDH = lactate dehydrogenase; TTH = tension-type headache.

Supplementary Table 5. Principal Component Analysis results of migraine symptoms (categorical variables).

	PC1	PC2	PC3	PC4
Migraine symptoms + LDH-Glomerular filtration				
**Relative contribution (%)**				
LDH values on admission	9.8	32.3	3.5	0.2
Glomerular filtration on admission	19.3	16.8	11.8	0.0
LDH worst values	8.1	32.3	6.8	1.2
Glomerular filtration worst values	21.4	16.6	4.9	0.0
**Coefficient**				
LDH values on admission	0.003	0.006	0.002	-0.000
Glomerular filtration on admission	0.032	-0.030	0.025	-0.001
LDH worst values	0.003	0005	0.002	-0.001
Glomerular filtration worst values	0.028	-0.025	0.014	-0.001
Migraine symptoms + CRP, PCT, D-dimer and lymphocyte on admission				
**Relative contribution (%)**				
CRP values on admission	38.1	8.9	0.4	0.0
PCT values on admission	32.5	12.8	1.8	0.6
D-dimer values on admission	0.1	0.1	28.1	14.4
Lymphocyte count on admission	4.7	0.2	20.0	25.0
**Coefficient**				
CRP values on admission	$9.21 \cdot 10^{-3}$	$4.45 \cdot 10^{-3}$	$0.96 \cdot 10^{-3}$	$0.24 \cdot 10^{-3}$
PCT values on admission	3.25	2.04	0.78	0.44
D-dimer values on admission	$-0.03 \cdot 10^{-3}$	$0.03 \cdot 10^{-3}$	$-0.71 \cdot 10^{-3}$	$0.51 \cdot 10^{-3}$
Lymphocyte count on admission	$-0.20 \cdot 10^{-3}$	$0.04 \cdot 10^{-3}$	$0.42 \cdot 10^{-3}$	$0.47 \cdot 10^{-3}$
Migraine symptoms + CRP, PCT, D-dimer and lymphocyte worst values				

**Relative contribution (%)**				
CRP worst values	40.9	5.8	0.0	0.4
PCT worst values	26.6	6.9	2.8	12.8
D-dimer worst values	4.8	6.6	18.9	11.6
Lymphocyte worst count	5.5	1.4	22.3	0.2
**Coefficient**				
CRP worst values	$7.94 \cdot 10^{-3}$	$2.98 \cdot 10^{-3}$	$-0.14 \cdot 10^{-3}$	$0.75 \cdot 10^{-3}$
PCT worst values	2.68	1.36	-0.87	1.86
D-dimer worst values	$0.07 \cdot 10^{-3}$	$0.09 \cdot 10^{-3}$	$0.15 \cdot 10^{-3}$	$-0.12 \cdot 10^{-3}$
Lymphocyte worst count	$-0.24 \cdot 10^{-3}$	$0.12 \cdot 10^{-3}$	$0.49 \cdot 10^{-3}$	$0.04 \cdot 10^{-3}$

PC = Principal Component. Migraine symptoms were unilateral, pulsating, and intense pain, aggravation by physical activity, nausea, and simultaneous phonophobia and photophobia. Continuous variables were the lactate dehydrogenase (LDH) and glomerular filtration levels (first and worst levels), C-reactive protein (CRP), procalcitonin (PCT), D-dimer and lymphocyte levels (on admission and worst levels)

Supplementary Table 6. Significant univariate logistic regression models of headache in the frontal area and pressing pain in patients hospitalized because of COVID-19.

	Odds Ratio and 95% CI	P-value	Missing
Headache in the frontal area			
Unilateral pain	4.45 [1.39, 13.99]	0.010	0
Pattern change	4.01 [1.32, 15.03]	0.021	0
Diffuse pain	5.65 [1.96, 17.90]	0.002	0
**Bilateral diffuse pain**	14.00 [3.67, 92.22]	< 0.001	0
Fatigue (COVID-19 symptom)	3.42 [1.18, 11.43]	0.030	0
Leukocyte count on admission	0.9997 [0.9995, 0.9999]	0.010	0
Lymphocyte count on admission	0.9990 [0.9981, 0.9998]	0.031	0
Platelet count on admission	0.9999 [0.9999, 0.9999]	0.026	2
Worst leukocyte count	0.9999 [0.9997, 0.9999]	0.033	1
**Abnormal IL-6 levels**	5.34 [1.87, 16.30]	0.002	0
Headache with infection	3.83 [1.26, 14.36]	0.027	0
**Autonomous symptoms**	0.18 [0.03, 1.03]	0.045	0
Pressing pain			
**Headache intensity**	0.53 [0.37, 0.73]	< 0.001	0
Disability caused by headache	0.97 [0.95, 0.99]	0.003	0
**Treatment resistant (analgesics)**	0.30 [0.10, 0.96]	0.038	0
Mild to moderate pain	4.07 [1.40, 14.86]	0.017	0
**Headache when sleeping**	0.24 [0.08, 0.70]	0.009	0
**Worst headache experienced in life**	0.27 [0.10, 0.66]	0.005	0
Head in left hemisphere vs. holocranial	0.22 [0.05, 0.82]	0.023	0
**Clinophilia**	0.32 [0.11, 0.83]	0.025	0

Variables in bold were included in the multivariate model. IL-6 = interleukin-6.

Supplementary Table 7. Principal Component Analysis results of quality of headache (categorical variables), and topography-laterality of headache (categorical variables).

	PC1	PC2	PC3	PC4
Quality of pain + CRP, PCT, D-dimer and lymphocyte on admission				
**Relative contribution (%)**				
CRP values on admission	33.9	14.3	0.1	0.0
PCT values on admission	30.9	17.0	0.1	1.4
D-dimer values on admission	0.2	0.1	42.9	8.7
Lymphocyte count on admission	5.4	0.6	16.5	32.6
**Coefficient**				
CRP values on admission	$8.68 \cdot 10^{-3}$	$5.64 \cdot 10^{-3}$	$0.43 \cdot 10^{-3}$	$-0.25 \cdot 10^{-3}$
PCT values on admission	3.17	2.35	0.18	0.69
D-dimer values on admission	$-0.05 \cdot 10^{-3}$	$-0.03 \cdot 10^{-3}$	$-0.88 \cdot 10^{-3}$	$0.40 \cdot 10^{-3}$
Lymphocyte count on admission	$-0.22 \cdot 10^{-3}$	$0.07 \cdot 10^{-3}$	$0.38 \cdot 10^{-3}$	$0.53 \cdot 10^{-3}$
Quality of pain + CRP, PCT, D-dimer and lymphocyte worst values				
**Relative contribution (%)**				
CRP worst values	29.3	19.1	0.2	0.2
PCT worst values	30.8	8.0	3.0	6.5
D-dimer worst values	2.8	6.2	47.0	8.3
Lymphocyte worst count	0.0	12.3	24.2	16.0
**Coefficient**				
CRP worst values	$6.72 \cdot 10^{-3}$	$5.42 \cdot 10^{-3}$	$0.50 \cdot 10^{-3}$	$0.57 \cdot 10^{-3}$
PCT worst values	2.89	1.47	-0.91	1.33
D-dimer worst values	$0.06 \cdot 10^{-3}$	$0.08 \cdot 10^{-3}$	$0.23 \cdot 10^{-3}$	$-0.10 \cdot 10^{-3}$
Lymphocyte worst count	$-0.02 \cdot 10^{-3}$	$-0.36 \cdot 10^{-3}$	$0.51 \cdot 10^{-3}$	$0.41 \cdot 10^{-3}$
Topography-laterality of headache + Platelet levels				
**Relative contribution (%)**				
Leukocyte count on admission	24.2	11.5	0.2	0.2
Lymphocyte count on admission	17.8	0.5	0.7	9.8

Platelet count on admission	14.9	4.4	0.2	27.0
Leukocyte worst count	19.6	3.8	0.2	8.3
**Coefficient**				
Leukocyte count on admission	$1.94 \cdot 10^{-4}$	$1.33 \cdot 10^{-4}$	$0.16 \cdot 10^{-4}$	$-0.17 \cdot 10^{-4}$
Lymphocyte count on admission	$3.93 \cdot 10^{-4}$	$0.67 \cdot 10^{-4}$	$0.79 \cdot 10^{-4}$	$2.92 \cdot 10^{-4}$
Platelet count on admission	$4.70 \cdot 10^{-6}$	$2.56 \cdot 10^{-6}$	$0.51 \cdot 10^{-6}$	$-6.34 \cdot 10^{-6}$
Leukocyte worst count	$9.05 \cdot 10^{-5}$	$3.97 \cdot 10^{-5}$	$-0.91 \cdot 10^{-5}$	$5.90 \cdot 10^{-5}$
Topography-laterality of headache + CRP, PCT, D-dimer and lymphocyte on admission				
**Relative contribution (%)**				
CRP values on admission	43.7	1.7	0.5	2.4
PCT values on admission	43.0	3.8	1.3	0.0
D-dimer values on admission	0.1	2.6	59.3	10.2
Lymphocyte count on admission	4.3	13.0	0.4	7.6
**Coefficient**				
CRP values on admission	$9.87 \cdot 10^{-3}$	$1.96 \cdot 10^{-3}$	$1.09 \cdot 10^{-3}$	$-2.34 \cdot 10^{-3}$
PCT values on admission	3.74	1.11	0.64	0.10
D-dimer values on admission	$-0.04 \cdot 10^{-3}$	$-0.22 \cdot 10^{-3}$	$1.03 \cdot 10^{-3}$	$0.43 \cdot 10^{-3}$
Lymphocyte count on admission	$-0.19 \cdot 10^{-3}$	$0.34 \cdot 10^{-3}$	$0.06 \cdot 10^{-3}$	$0.26 \cdot 10^{-3}$
Topography-laterality of headache + CRP, PCT, D-dimer and lymphocyte worst values				
**Relative contribution (%)**				
CRP values on admission	41.7	4.2	0.3	0.2
PCT values on admission	34.1	2.6	7.0	0.0
D-dimer values on admission	4.6	7.8	37.9	6.8
Lymphocyte count on admission	7.3	8.0	3.0	61.5
**Coefficient**				
CRP values on admission	$8.02 \cdot 10^{-3}$	$2.56 \cdot 10^{-3}$	$0.68 \cdot 10^{-3}$	$-0.61 \cdot 10^{-3}$

PCT values on admission	3.03	0.83	-1.38	0.17
D-dimer values on admission	$0.07 \cdot 10^{-3}$	$0.09 \cdot 10^{-3}$	$0.21 \cdot 10^{-3}$	$0.09 \cdot 10^{-3}$
Lymphocyte count on admission	$-0.28 \cdot 10^{-3}$	$0.29 \cdot 10^{-3}$	$-0.18 \cdot 10^{-3}$	$0.81 \cdot 10^{-3}$

PC = Principal Component. Analyzed quality of headache was pressing, pulsating, and stabbing pain. Topography-laterality was frontal, temporal, periocular, and bilateral diffuse pain. Continuous variables were C-reactive protein (CRP), procalcitonin (PCT), D-dimer and lymphocyte levels (on admission and worst levels), and platelet levels (on admission and worst values, only for topography-laterality of headache).

Supplementary Table 8. Principal Component Analysis results of the C-reactive protein (CRP), procalcitonin (PCT), D-dimer and lymphocyte levels (on admission and worst values; continuous variables), and most frequent symptoms (categorical variables).

	PC1	PC2	PC3	PC4
Most frequent symptoms + CRP, PCT, D-dimer and lymphocyte on admission				
**Relative contribution (%)**				
CRP values on admission	45.3	0.1	0.3	2.4
PCT values on admission	44.2	1.1	2.0	1.4
D-dimer values on admission	0.2	15.5	19.7	2.2
Lymphocyte count on admission	3.4	0.9	35.9	0.7
**Coefficient**				
CRP values on admission	0.10	$0.53 \cdot 10^{-3}$	$0.87 \cdot 10^{-3}$	$-2.29 \cdot 10^{-3}$
PCT values on admission	3.79	0.59	0.81	0.68
D-dimer values on admission	$-0.06 \cdot 10^{-3}$	$0.53 \cdot 10^{-3}$	$-0.59 \cdot 10^{-3}$	$-0.20 \cdot 10^{-3}$
Lymphocyte count on admission	$-0.17 \cdot 10^{-3}$	$0.09 \cdot 10^{-3}$	$0.56 \cdot 10^{-3}$	$0.08 \cdot 10^{-3}$
Most frequent symptoms + CRP, PCT, D-dimer and lymphocyte worst values				
**Relative contribution (%)**				
CRP values on admission	46.1	0.1	1.7	0.1
PCT values on admission	38.4	2.2	4.0	2.1
D-dimer values on admission	6.1	0.1	52.8	0.8
Lymphocyte count on admission	4.3	18.6	1.9	26.8
**Coefficient**				
CRP values on admission	$8.42 \cdot 10^{-3}$	$-0.38 \cdot 10^{-3}$	$1.60 \cdot 10^{-3}$	$-0.42 \cdot 10^{-3}$
PCT values on admission	3.22	0.77	-1.04	0.75
D-dimer values on admission	$0.08 \cdot 10^{-3}$	$-0.01 \cdot 10^{-3}$	$0.24 \cdot 10^{-3}$	$-0.03 \cdot 10^{-3}$
Lymphocyte count on admission	$-0.22 \cdot 10^{-3}$	$0.45 \cdot 10^{-3}$	$0.14 \cdot 10^{-3}$	$0.54 \cdot 10^{-3}$

PC = Principal Component. Most frequent symptoms were sensitivity to stimuli, pressing pain, intense pain, and frontal/bilateral diffuse pain.

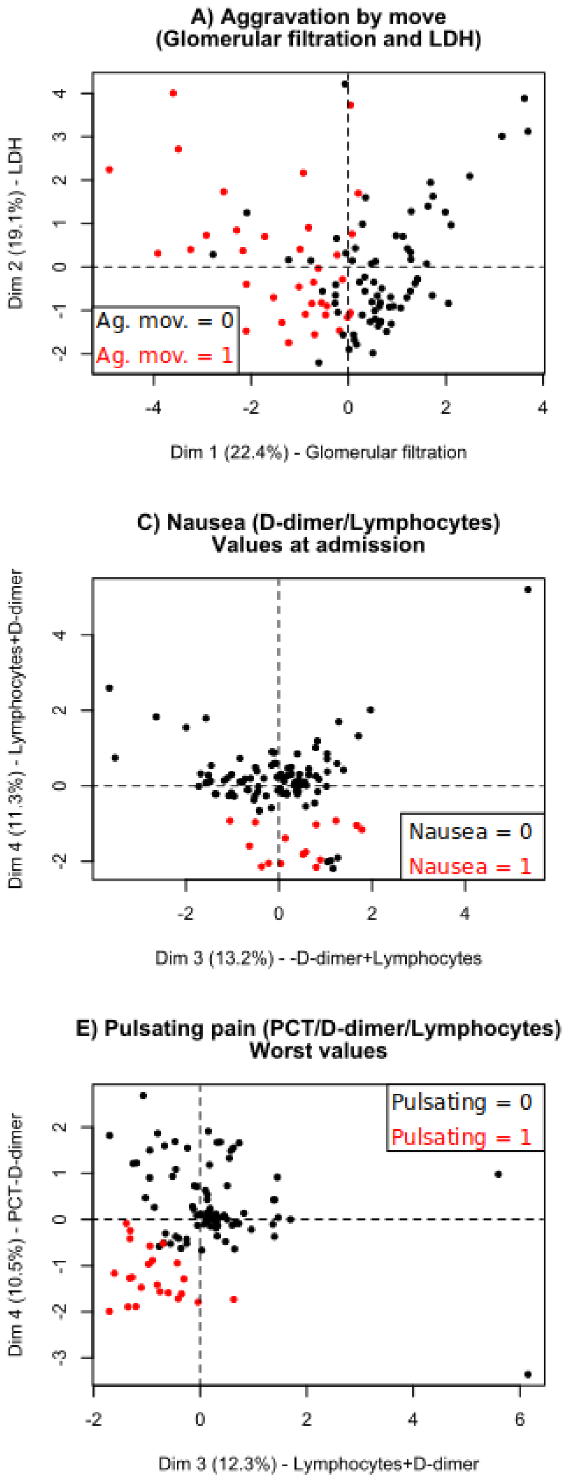

Supplementary Figure 8. Mix PCA of results from laboratory tests (LDH and glomerular filtration worst levels and levels at admission in A), and diverse migraine symptoms. X- and Y-axis contain the values of two principal components. 0 = no symptom; 1 = present symptom.

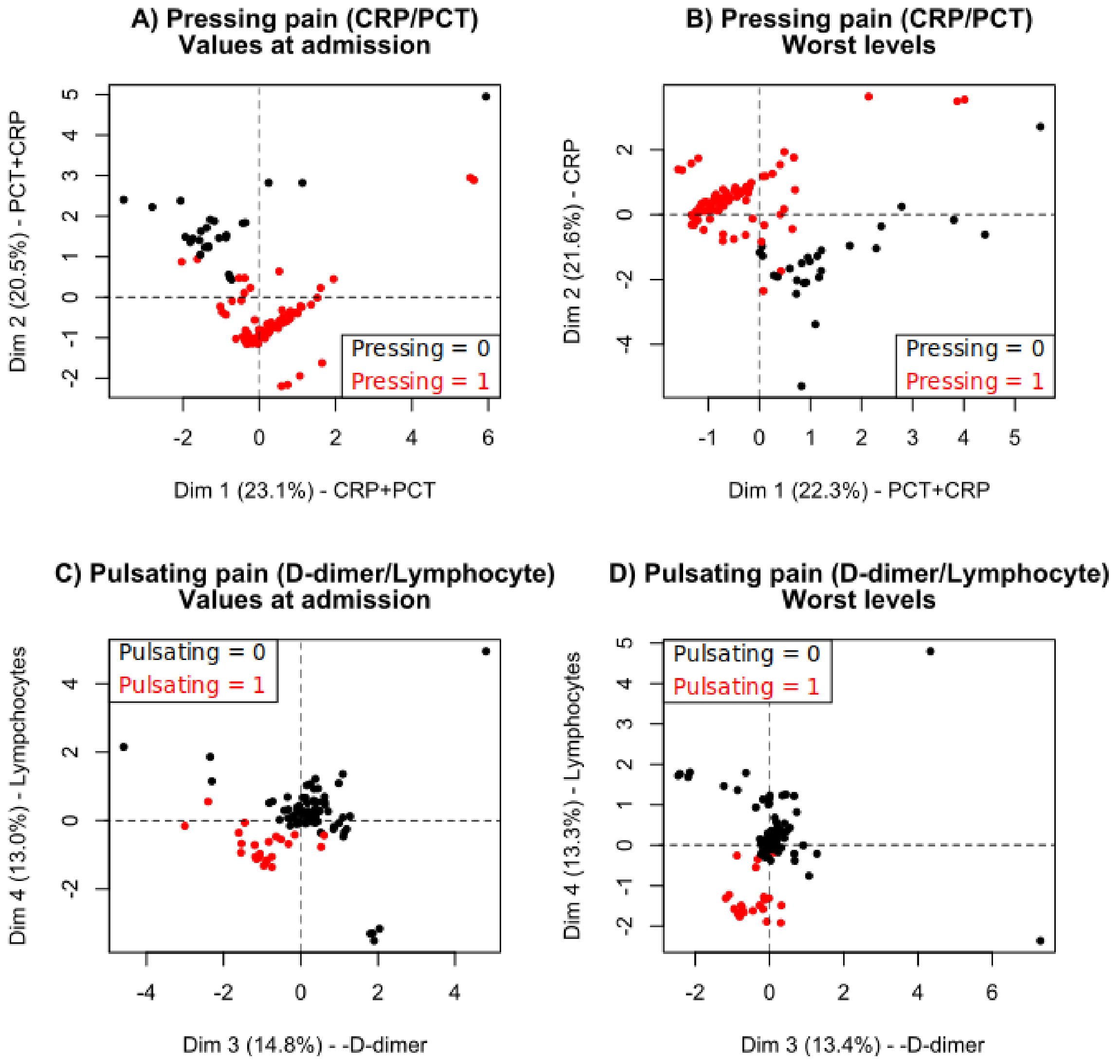

Supplementary Figure 9. Mix PCA of results from laboratory tests and quality of pain. X- and Y-axis contain the values of two principal components. 0 = no quality; 1 = present quality.

A) Pain in frontal area

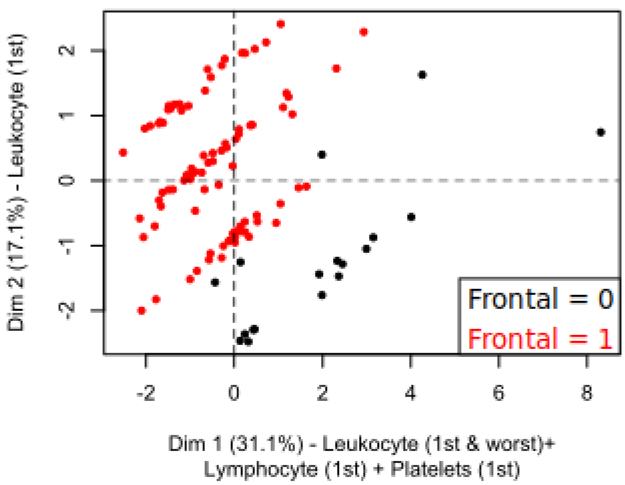

B) Pain in periocular area

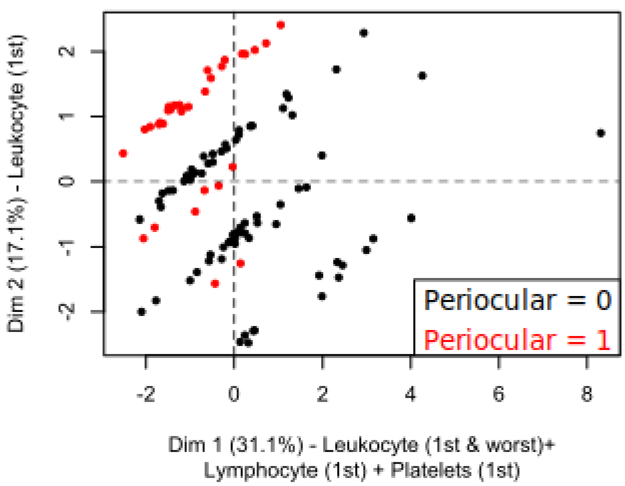

C) Bilateral diffuse pain

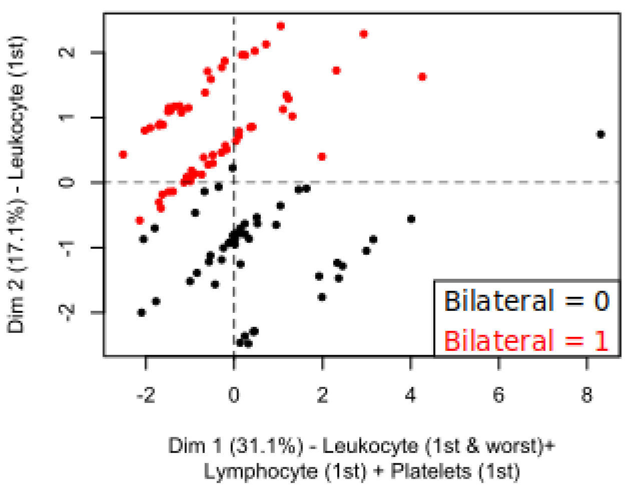

Supplementary Figure 10. Mix PCA of results from laboratory tests (leukocytes, lymphocytes, and platelets levels at admission, and worst platelet levels) and topography and laterality of pain. X- and Y-axis contain the values of the two first principal components. 0 = absent topology/laterality; 1 = present topology/laterality.

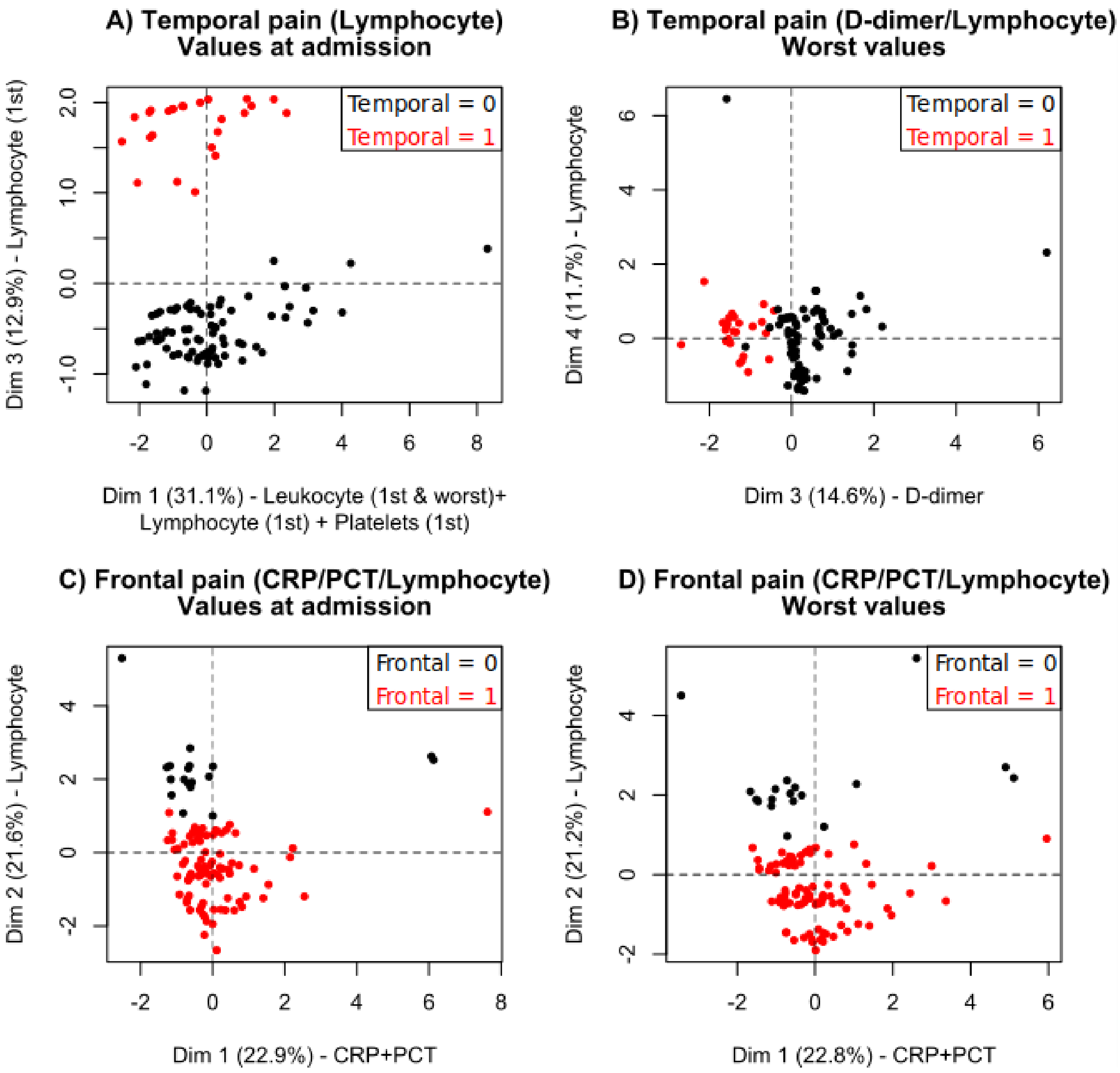

Supplementary Figure 11. Mix PCA of results from laboratory tests and topography of pain. X- and Y-axis contain the values of two principal components. 0 = absent topology; 1 = present topology.

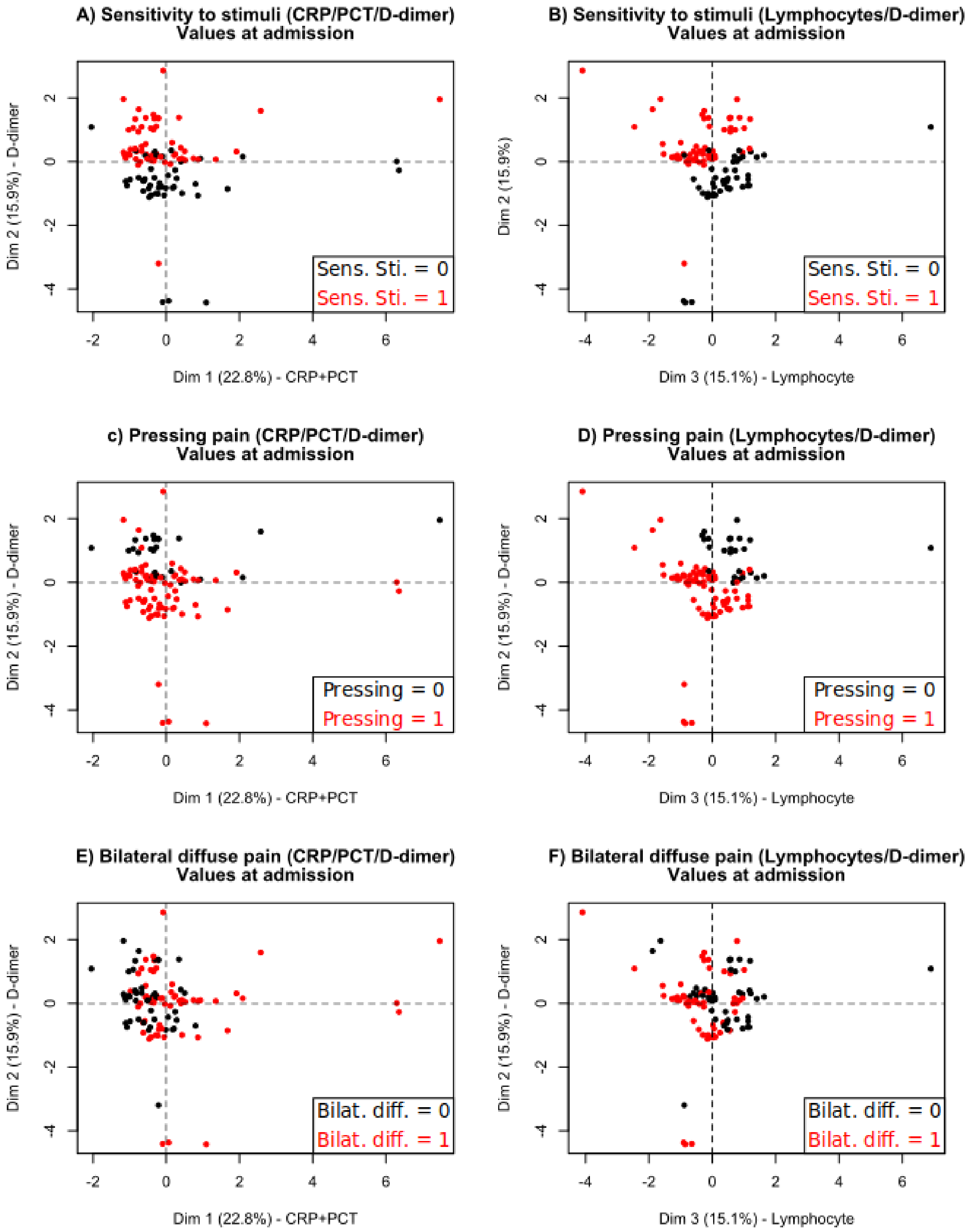

Supplementary Figure 12. Mix PCA of results from laboratory tests at admission and most frequent headache characteristics. X- and Y-axis contain the values of two principal components. 0 = absent characteristic; 1 = present characteristic.

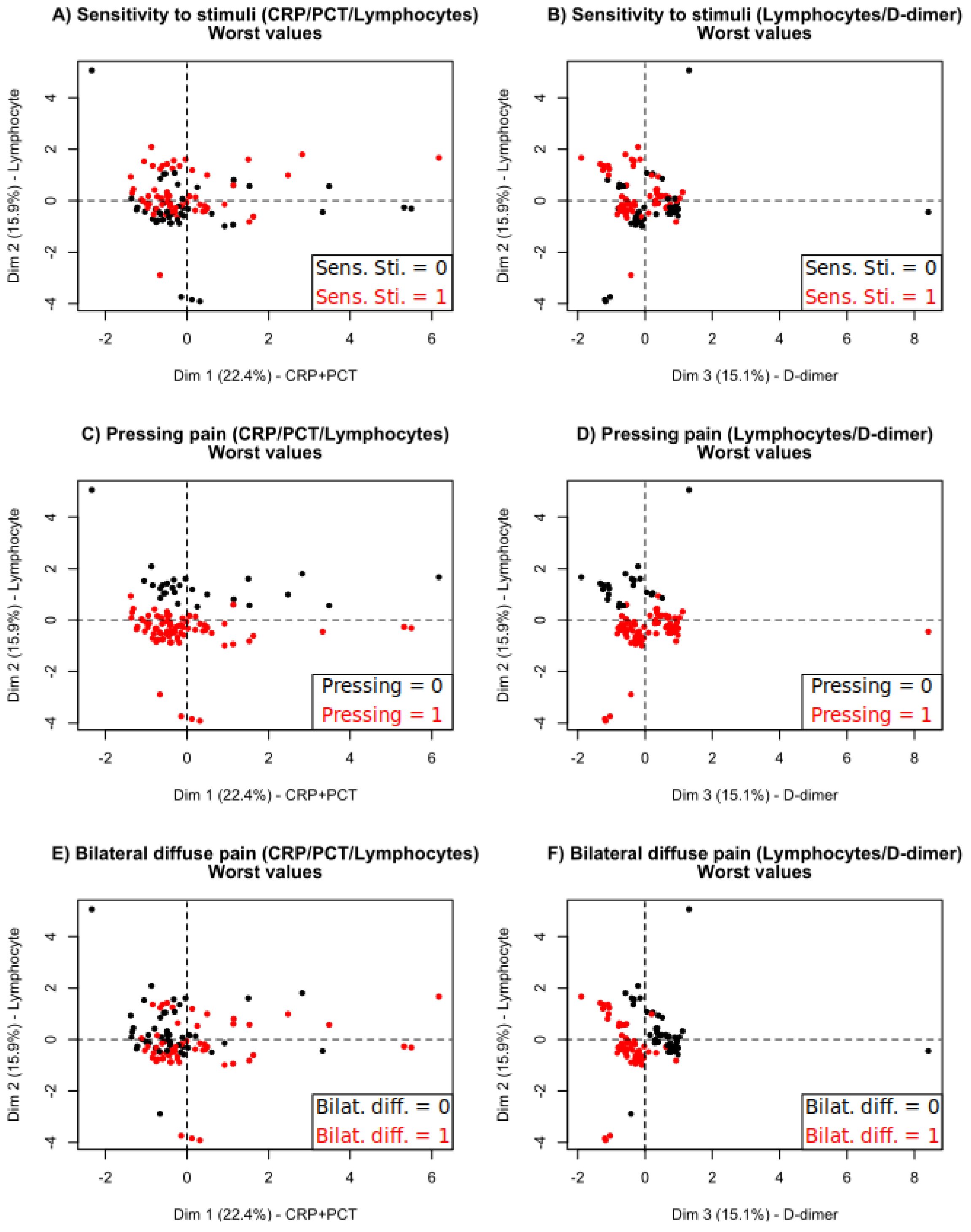

Supplementary Figure 13. Mix PCA of results from worst laboratory tests and most frequent headache characteristics. X- and Y-axis contain the values of two principal components. 0 = absent characteristic; 1 = present characteristic.
